# 2D materials-based next-generation multidimensional photodetectors

**DOI:** 10.1038/s41377-025-01995-8

**Published:** 2025-10-10

**Authors:** Jiayue Han, Ziyi Fu, Jingxuan Wei, Song Han, Wenjie Deng, Fangchen Hu, Zhen Wang, Hongxi Zhou, He Yu, Jun Gou, Jun Wang

**Affiliations:** 1 School of Optoelectronic Science and Engineering, University of Electronic Science and Technology of China, Chengdu, 611731 China; 2 Innovative Institute of Electromagnetic Information and Electronic Integration, College of Information Science & Electronic Engineering, Zhejiang University, Hangzhou, 310027 China; 3 Key Laboratory of Optoelectronics Technology, Ministry of Education, Faculty of Information Technology, Beijing University of Technology, Beijing, 100124 China; 4 Zhangjiang Laboratory, Shanghai, 201204 China; 5 State Key Laboratory of Infrared Physics, Shanghai Institute of Technical Physics Chinese Academy of Sciences, Shanghai, 200003 China

**Keywords:** Imaging and sensing, Optical sensors

## Abstract

With the rapid advancement of the information age, the demand for multi-dimensional light information detection has significantly increased. Traditional Fourier-transform infrared (FTIR) spectrometers and pooptical power, andlarimeters, due to their bulky structure, are no longer suitable for emerging fields such as medical diagnostics, secure communications, and autonomous driving. As a result, there is a pressing need to develop new miniaturized on-chip devices. The abundant two-dimensional (2D) materials, with their unique light-matter interactions, offer the potential to construct high-dimensional spatial mappings of incident light, paving the way for the development of novel ultra-compact multi-dimensional deep optical sensing technologies. Here, we review the interconnections of multi-dimensional information and their relationship with 2D materials. We then focus on recent advances in the development of novel dimensional detectors based on 2D materials, covering dimensions such as intensity, time, space, polarization, phase angle, and wavelength. Furthermore, we discuss cutting-edge technologies in multi-dimensional fusion detection and highlight future technological prospects, with a particular emphasis on on-chip integration and future development.

## Abstract (web summary)

This review highlights the advancement of multidimensional detection in 2D materials. It encompasses six novel dimensionalities and hybrid-dimensional photodetectors, offering guidance for developing next-generation multidimensional photodetectors.

## Graphical abstract

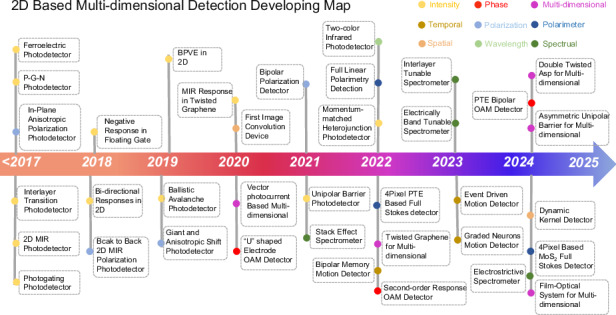

## Introduction

Photoelectric multi-dimensional detectors represent a class of sensors that integrate optical and electrical properties, enabling the acquisition and processing of multi-dimensional information^1–4^. These devices hold significant potential for diverse applications across various fields, including remote sensing and environmental monitoring, life sciences, national defense and security, deep space astronomy, and intelligent driving^5–7^. Natural light contains a wealth of multi-dimensional information, which, when properly understood, can offer comprehensive insights into complex optical phenomena. Efficient extraction and reconstruction of such multi-dimensional information from light is crucial but remains a formidable challenge. Multi-dimensional information encompasses several dimensions, including intensity, spectrum, polarization, phase angle, time, and position. Traditional approaches to multi-dimensional detection often require the integration of complex optical components within spatial or temporal domains, leading to bulky systems with complicated operation protocols that hinder practical deployment^8,9^. Furthermore, conventional detectors often struggle with extracting mixed multi-dimensional information due to cross-talk and the compression-induced loss of critical data, which impedes their ability to meet the detection demands of multi-dimensional information^8,10^. Therefore, accurately separating overlapping information within a single device, through advancements in engineering and materials science, is a key technological challenge. The emergence of novel materials, particularly those ranging from 0D to 3D, has brought new hope for the design of multi-dimensional detectors. However, 3D and 0D materials often lose the ability to detect critical information, such as polarization and phase angle, due to their inability to form single crystals. In addition, complex multi-dimensional composite information is difficult to detect using topologically trivial materials. In contrast, two-dimensional materials (including quasi-one-dimensional materials) with superior optoelectronic and physical properties enabled by their rich material systems offer compact and efficient solutions for the design of advanced ultra-compact spatiotemporal integrated detectors^11–15^. Research of 2D multi-dimensional detection has thus evolved into a highly interdisciplinary field that combines device engineering, materials science, physics, and machine learning^16^.

**Fig. 1:**
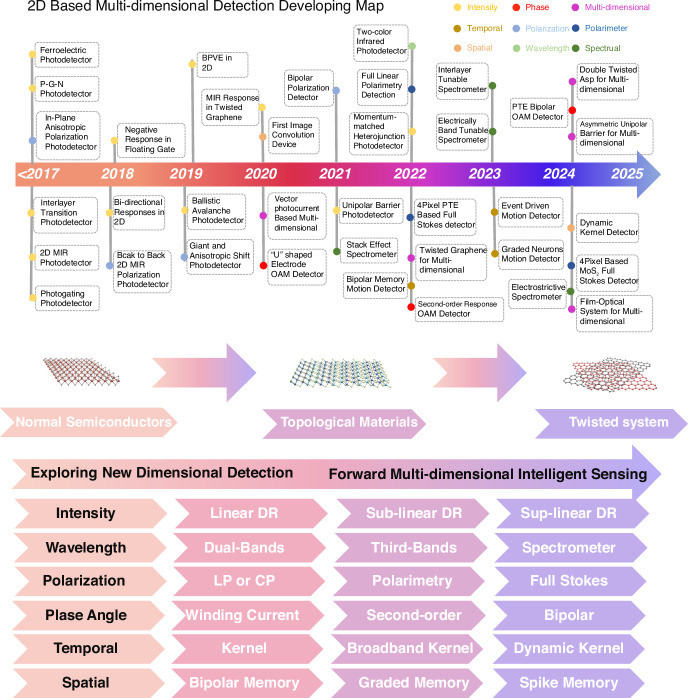
Time developing line of multi-dimensional detection based on 2D materials

The 2D materials library offers a diverse range of materials, including semiconductors, semi-metals, and insulators, characterized by broad band structures spanning ultraviolet to terahertz wavelengths^13,14,17–24^. These materials also exhibit complex polarization states and a wide range of possible band alignments, making them an ideal platform for the design of sophisticated optoelectronic devices^11,25,26^. Early research focused on leveraging the unique properties of 2D materials to develop new detectors optimized for specific dimensions (Fig. 1). For example, unipolar barrier^27^, ballistic tunneling^28^, and photogating effects^29^ have been utilized to design intensity detectors, while multi-heterojunctions have enabled the development of wavelength detectors capable of operating in dual or triple bands^30,31^. To achieve miniaturization and facilitate chip-level integration, recent advancements have led to the development of on-chip polarization detectors and compact spectrometers^32–36^. In particular, algorithms have played an increasingly crucial role in compact spectrometers, where the use of advanced neural network algorithms, such as convolutional neural networks (CNN) and deep neural networks (DNN), has enabled high-precision reconstruction of novel multi-dimensional information^10,37^.

Furthermore, the human visual system provides a valuable reference for designing detectors that optimize performance across various dimensions^38,39^, such as spectral detection (RGB detection)^40,41^, temporal detection (motion detection)^42,43^, and spatial detection (edge detection, depth-of-field detection)^44,45^. However, the wavelength range of the human eye is limited (400-700 nm), and its inability to detect polarization, phase angle, and other information constrains the development of eye-inspired multi-dimensional detectors^46^. Inspired by the complex retinal structure of the mantis shrimp, which utilizes specialized horizontal cells (R1-R8) to simultaneously detect polarization states and color variations, such a design could decode highly entangled optical information^47,48^. Therefore, studying the visual systems of various organisms holds potential for further optimizing the functional integration of multi-dimensional detection devices. Early investigations into multi-dimensional detection began with the use of angle-dependent birefringent materials to detect polarization states, although these methods often required additional power calibration devices^49,50^. More advancements in three-terminal vector photonic current systems have enabled multi-dimensional detection (e.g., intensity and linear polarization) without the need for power calibration^51^. The continued progress in 2D materials and algorithms, such as topological Weyl semimetals^52–54^, moiré quantum system^55,56^, and graph neural networks (GNN)^57^, has led to new approaches for overcoming previous limitations. Additionally, the complex physical properties of these 2D materials, whether used in isolation or in combination with micro-nano structures, create a mapping bridge for linking multi-dimensional optical information with light responses in both linear and nonlinear light-matter interactions^37,58^. This mapping process can be inverted and solved using advanced neural network algorithms, thereby extending the scope of multi-dimensional detection^10^. Looking ahead, the development of highly compact, multifunctional, reconfigurable, and intelligent multi-dimensional detectors is poised to become a major area of research, promising significant advances in sensor technology.

Recently, multi-dimensional detection based on twisted 2D materials and 2D/metasurface structures has garnered significant attention, positioning it as a cutting-edge research direction for multi-functional vision sensing. This field has developed rapidly and now includes an initial framework of six primary detection dimensions (as illustrated in the lower half of Fig. 1). In this review, we first introduce new types of dimensional optoelectronic detectors based on 2D materials, covering intensity, spectral, polarization, phase angle, temporal, and spatial detectors. We then summarize the related performance parameters for each of these dimensions. Following this, we provide a detailed analysis and discussion of the working principles and technological progression of multi-dimensional fusion detectors. Finally, we highlight the future development of 2D material-based multi-dimensional detectors, particularly focusing on the impact of various parameters and integration challenges.

## Traditional bulk materials vs 2D materials for new dimensional detectors

**Fig. 2:**
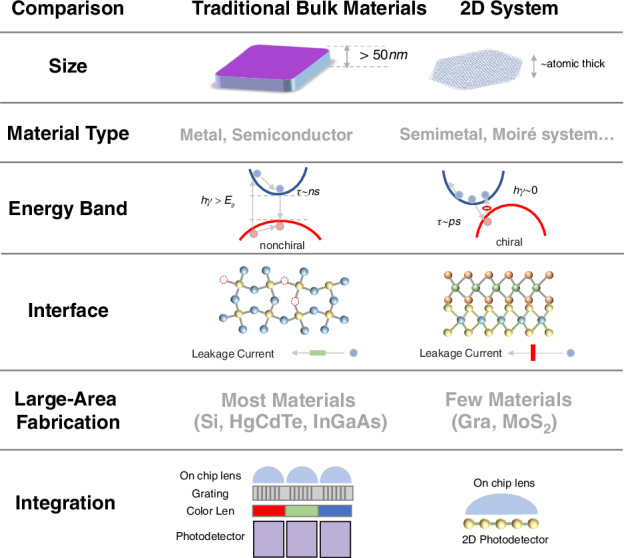
Comparative study of 2D materials vs. bulk materials for multi-dimensional sensing

With the advent of the big data era and the growing demand for full-light-field detectors, the development of novel-dimensional or multi-dimensional detectors has become increasingly imperative. 2D materials, with their atomic-scale thickness, hold significant promise for enabling ultra-compact device architectures, thereby facilitating portable multi-dimensional detection systems (Fig. 2). In contrast, conventional bulk materials are fundamentally constrained by their material categories and intrinsic band structures, necessitating complex optical systems (including splitters, diffraction gratings, and spectral filters) to achieve the required separation and analysis of physical light-field parameters such as polarization, spectrum, and phase. The inherent bulkiness of these traditional material systems combined with their auxiliary optics substantially limits their practical applications in emerging fields like automotive, biomedical, and remote sensing technologies.

The rich material systems of 2D materials, encompassing topological Weyl semimetals and moiré superlattices, offer unprecedented opportunities for coupling and mapping material properties with multi-dimensional light-field information. These systems enable effective separation of overlapping information including chirality, wavelength, and phase characteristics (Fig. 2). Furthermore, their unique carrier dynamics suggest exceptional capability for ultrafast process capture (with detection bandwidths reaching picosecond timescales). The van der Waals interfaces in 2D materials present additional performance advantages by their defect-free nature, whereas conventional bulk material interfaces inevitably suffer from substantial defect densities that lead to elevated dark currents and noise levels, ultimately compromising detection sensitivity. As aforementioned, 2D material-based detectors can leverage their intrinsic material properties to achieve multi-dimensional single-pixel integration, thereby minimizing the physical device count required for multi-dimensional pixel integration.

## Overview of multi-dimensional detectors

**Fig. 3:**
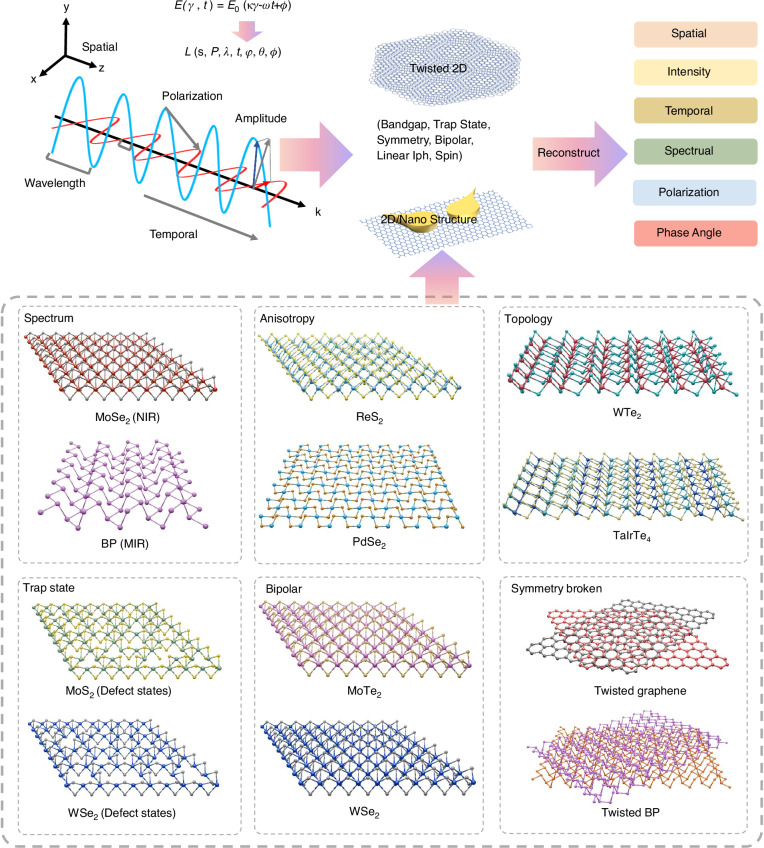
The connection between Multi-dimensional detection and 2D materials

Notably, light can be treated as an electromagnetic field for a more comprehensive analysis. To understand the multi-dimensional information structure of the light field, the electric field wave equation facilitates our understanding of the independent relationships between different dimensional parameters. According to the electric field wave equation:

$${\nabla }^{2}E-\frac{1}{{C}^{2}}\frac{{\partial }^{2}E}{\partial {t}^{2}}=0$$

the solution for a plane wave electric field is given by:

$${\rm{E}}\left(\vec{r},t\right)={E}_{0}\cos \left(\vec{k}\cdot \vec{r}-\omega t+\phi \right)$$

as shown in Fig. 3. Here, ${E}_{0}$ represents the intensity information of the light, $\vec{r}$ describes the spatial information of the light, and *t*-dependent parameters reflect the temporal variation of the wave. Additionally, the electric field equation also incorporates wavelength information of the light:$\,{\rm{\lambda }}={\rm{c}}/\omega$.This equation can be generally written as:

$${\rm{E}}\left(z,t\right)={E}_{0}\cos \left({kz}-\omega t+\phi \right)$$

where $\phi$ is the initial phase angle of the wavefront. If $\phi$ is constant, it indicates that the wavefront is a plane of equal phase. In common phase angle detectors, the analysis often extends to orbital angular momentum detectors, which detect complex phase vortex distributions. This is expressed by the following equation (in cylindrical coordinates):

$${\rm{E}}\left(R,\vartheta ,z,t\right)={E}_{0}\left(R,z\right)exp {{(}}{\mathcal{i}}{\mathcal{l}}\vartheta )\widehat{{e}_{\vartheta }}exp {{(}}{{-}}{\mathcal{i}}{kz}{\mathscr{+}}{\mathcal{i}}\omega t)$$

where ${\mathcal{l}}$ represents the topological charge of the vortex, which describes the number of phase rotations of the light beam, and $\exp ({\mathcal{i}}{\mathcal{l}}\vartheta )$ is the vortex phase factor. Light polarization is studied through the projections of the electric field components along the x- and y-axes:

$$\begin{array}{l}{\rm{E}}\left(z,t\right)={E}_{x0}\cos \left({kz}-\omega t+{\phi }_{x}\right)\hat{x}\\\qquad\qquad+{E}_{y0}\cos \left({kz}-\omega t+{\phi }_{y}\right)\hat{y}\end{array}$$

The polarization state is determined by the phase difference between ${\phi }_{x}$ and ${\phi }_{y}$. If the phases are identical, the light is linearly polarized. If the phase difference is ±π/2, the light is circularly polarized. Elliptical polarization is the generalization of both linear and circular polarization.

The plane wave electric field equation fundamentally represents the intrinsic multi-dimensional information of light. Low-dimensional materials, as platforms for multi-dimensional detection, are designed using various types of 2D materials (Fig. 3)^59,60^. The intensity dimension primarily depends on the alignment of the energy bands, with many 2D materials exhibiting thickness-dependent, tunable bandgap characteristics^61^. This enables the creation of high-performance type-II heterojunctions, quantum wells, and single-barrier energy band structures for intensity detection. The wavelength of light is often influenced by the material’s bandgap and structural dimensions. 2D materials span the entire spectrum from ultraviolet to terahertz, including visible light-transition metal dichalcogenides (TMDs) such as MoS_2_^62^, MoSe_2_^63^, WSe_2_^64^, WS_2_^65^; near-infrared MoTe_2_^66^, Te^67^; mid-infrared BP^27^, Asp^68^, PdSe_2_^69^, PdTe_2_^70^, and even new ternary materials (e.g., Ta_2_NiSe_5_^71^, Ta_2_NiS_5_^72^, Nb_2_GeTe_4_^73^, Nb_2_SiTe_4_^74^), as well as graphene, which covers the full wavelength range. In addition, 2D materials provide a wealth of materials that respond to polarization, including, ReS_2_^75^, ReSe_2_^76^, BP^77^, Asp^78^ and materials that generate circular polarization, such as Te^79^ and MoTe_2_^80^. Phase angle detectors require 2D materials with special symmetries, including type-II topological Weyl semimetals such as WTe_2_ and TaIrTe_4_^54,81,82^. Certain 2D material-based devices exhibit temporally tunable photoresponse characteristics, achieved through modulation of surface-bound states in TMDs. Representative systems such as MoS_2_ and WSe_2_ demonstrate programmable response weighting when subjected to ultraviolet illumination treatments^83,84^. Regarding spatial resolution and computational functionality, these are principally realized through ambipolar 2D semiconductors, including graphene, MoTe_2_, and WSe_2_. These materials possess the unique capability for electrically reconfigurable response polarity control, enabling dynamic switching between n-type and p-type photoconduction regimes^85^.

By employing 2D materials and composite structures, the response can be transformed, converting the high-dimensional input information into photogenerated current signals. These signals can then be processed using inverse operations to reconstruct the original high-dimensional information. The series of nonlinear light-matter interaction characteristics enabled by 2D material structures allows for the encoding of input information, generating complex mapping relationships.

### The figure of merit of new dimensional detectors

With emerging new dimensional photodetectors, a comprehensive discussion of multidimensional detector parameters becomes essential for evaluating the performance of these novel devices. Accordingly, we hereby provide formal definitions for the key parameters mentioned in this work to facilitate better understanding.

For intensity detectors, responsivity (*R*) serves as the fundamental guarantee of the detector’s sensitivity, and can therefore be expressed as:

$$R=\frac{{I}_{{ph}}}{P}=\frac{\eta q\lambda }{{hc}}$$

Where ${I}_{{ph}}$ denotes the photocurrent, $P$ represents the optical power, $\eta$ is the quantum efficiency, $q$ is the elementary charge, and $\lambda$ is the wavelength. Accordingly, the specific detectivity can be expressed as:

$${D}^{* }=\frac{R\sqrt{{Af}}}{{i}_{{noise}}}$$

Where $A$ denotes the effective photosensitive area of the detector, $f$ is the operational bandwidth, and ${i}_{{noise}}$ represents the total noise current. The noise level and the saturated photocurrent jointly determine the operating range of an intensity detector, commonly referred to as the linear dynamic range (LDR). The LDR defines the maximum range over which the output signal of the detector maintains a linear relationship with the incident optical power, and is given by:

$${LDR}=10\,{log }_{10}\left(\frac{{I}_{\max }}{{I}_{\min }}\right){\rm{or}}\,20\,{log }_{10}\left(\frac{{I}_{\max }}{{I}_{\min }}\right)$$

Where ${I}_{\max }$ is the saturated optical intensity and ${I}_{\min }$ is the noise-equivalent optical intensity.

For polarization detectors, the polarization ratio (${PR}$) is a dimensionless parameter used to describe the polarization state of an electromagnetic wave. It is defined as the intensity ratio between two orthogonal polarization components, commonly expressed as:

$${PR}=\frac{{I}_{{LP}-\max }}{{I}_{{LP}-\min }}$$

Where ${I}_{{LP}-\max }$ and ${I}_{{LP}-\min }$ represent the intensities of two orthogonal linearly polarized components, corresponding to the maximum and minimum intensity positions, respectively. In recent developments, the polarization ratio (PR) has been extended to include negative values. A PR of -1 enables certain performance enhancements in multidimensional fusion detectors that integrate intensity and polarization detection. In the context of circularly polarized light detection or spin-resolved photodetection, the g-factor serves as a key parameter that quantifies the difference in detector response between left-handed and right-handed circularly polarized light. It is defined as:

$${\rm{g}}=\frac{{I}_{{RCP}}-{I}_{{LCP}}}{{I}_{{RCP}}+{I}_{{LCP}}}$$

Where ${I}_{{RCP}}$ and ${I}_{{LCP}}$ denote the photocurrent signals corresponding to right-handed and left-handed circularly polarized light, respectively. For more complex polarization states of light, the full Stokes parameters are typically used. This set of four parameters (${S}_{0}$,$\,{S}_{1}$,$\,{S}_{2}$,$\,{S}_{3}$) provides a complete description of the polarization state of light. These parameters are defined as follows:

$${S}_{0}={I}_{0^\circ }+{I}_{90^\circ }$$

$${S}_{1}={I}_{0^\circ }-{I}_{90^\circ }$$

$${S}_{2}={I}_{45^\circ }-{I}_{135^\circ }$$

$${S}_{3}={I}_{{RCP}}-{I}_{{LCP}}$$

Among them, ${S}_{0}$ represents the total intensity of the light, ${S}_{1}$ denotes the intensity difference between horizontal and vertical linear polarization components, ${S}_{2}$ corresponds to the intensity difference between linear polarization components at 45° and 135°, and ${S}_{3}$ represents the intensity difference between right-handed and left-handed circular polarization components. In addition, we can estimate the degree of linear polarization (DoLP) and angle of linear polarization (AoLP) as follows:

$${\rm{DoLP}}=\frac{\sqrt{{S}_{1}^{2}+{S}_{2}^{2}}}{{S}_{0}}$$

$${\rm{AoLP}}=\frac{1}{2}\arctan \left(\frac{{S}_{2}}{{S}_{1}}\right)\left({\rm{when}}\,{S}_{1} > 0\right){\rm{or}}\frac{1}{2}\arctan \left(\frac{{S}_{2}}{{S}_{1}}\right)+\frac{\pi }{2}\left({\rm{when}}\,{S}_{1} < 0\right)$$

For spectral detectors, miniaturization typically characterized by the effective area $A$ and spectral resolution are of particular importance. The spectral resolution is defined as:

$$R=\frac{\lambda }{\Delta \lambda }$$

Where $\Delta \lambda$ represents the minimum wavelength interval that allows the spectrometer to resolve adjacent spectral peaks, and $\lambda$ denotes the operating wavelength.

For spatial detectors, the resolution is influenced by the pixel size of the array matrix. Typically, the highest spatial frequency that the sensor can resolve (measured in line pairs per millimeter) is given by:

$${\gamma }_{\max }=\frac{1}{2p}$$

Where $p$ denotes the pixel size.

For temporal-motion detectors, the device response speed largely determines the achievable frame rate. Response time ($\tau$) refers to the duration required for the detector to produce a stable output signal after illumination, including both the rise time (${\tau }_{{rise}}$) and fall time (${\tau }_{{fall}}$). The frame rate (${f}_{{frame}}$) which is expressed in frames per second (FPS) represents the number of image frames the detector can capture per second, thereby defining the temporal resolution. High frame rates are essential for observing fast transient processes. The maximum allowable frame rate is given by:

$${f}_{{frame},\max }\approx \frac{1}{\tau }$$

For orbital angular momentum (OAM) detectors, a key performance metric is the minimum resolvable spacing between adjacent OAM modes (topological charge ${\mathcal{l}}$). A higher mode resolution enables simultaneous detection of a greater number of OAM states. This parameter is defined by the following equation:

$$\Delta {\mathcal{l}}={{\mathcal{l}}}_{\max }-{{\mathcal{l}}}_{\min }+1$$

Here,$\,{{\mathcal{l}}}_{\max }$ and ${{\mathcal{l}}}_{\min }$ denote the maximum and minimum topological charges supported by the detector, respectively.

### Improved performance of various dimensional detectors

**Fig. 4:**
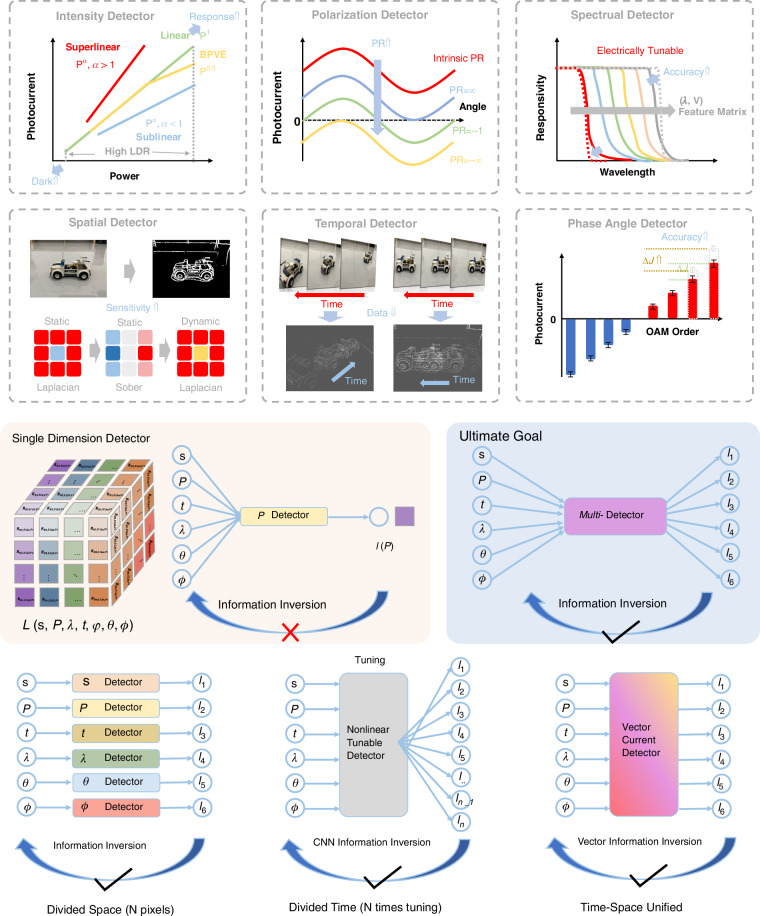
The metrics and general design strategies for multi-dimensional detectors

With the development in recent years, information-dimensional detectors have been widely researched and explored. Leveraging the latest advancements in two-dimensional materials, heterojunction energy band regulation, and structural design characteristics, these detectors can achieve performance optimization in specific dimensions, paving the way for practical applications. Here, we introduce the development trends and performance optimizations of six types of novel dimensional detectors (Fig. 4):

1. **Intensity Detector**: As the most fundamental scalar detector, the intensity detector plays a crucial role, particularly in power calibration, where the detector’s response is typically required to exhibit a certain linear relationship with power. Here, we outline three common relationships between the detector’s photoelectric response and input power: linear, sublinear, and superlinear. Additionally, there exists a special type of nonlinear relationship dominated by the bulk photovoltaic effect (BPVE), which enables higher photovoltaic efficiency. Researchers aim to achieve the maximum linear dynamic range by enhancing noise suppression and improving photoresponse performance.
2. **Polarization Detector**: Polarization detectors, including linear polarization and circular polarization detectors, are realized in two-dimensional materials through anisotropic and chiral structures. The polarization ratio (PR) and the g-factor are critical quality factors. Since 2021, the emergence of positive and negative polarization ratios has significantly enhanced polarization performance, theoretically achieving a PR of positive and negative infinity. Researchers have further discovered that when the PR is -1, power calibration can be omitted in multi-dimensional detection. Meanwhile, a full-Stokes detector typically requires four-parameter measurements to determine the complete polarization state.
3. **Spectral Detector**: 2D-based spectral detectors have evolved from dual-band detectors to today’s computational spectral detectors, becoming increasingly mature. These detectors can accurately resolve subtle changes in the input spectrum and reconstruct it. Both dual-band detectors and computational spectral detectors are capable of tuning their response spectra through electrical modulation. However, spectral crosstalk induced by this modulation often becomes a critical factor limiting the precision of spectral detectors. Leveraging more sophisticated physical mechanisms and device structures holds promise for mitigating this crosstalk and enhancing detection accuracy.
4. **Spatial Detector**: Spatial detectors can further optimize the input’s 2D visual intensity distribution through linear and nonlinear operations, such as the edge enhancement function, which is crucial for human vision. Nonlinear responses can further improve the feature-to-noise ratio, while linear responses typically simulate edge enhancement effects. From the initial Laplacian static operator in kernels to the current dynamic Laplacian operator with feedback, the nonlinear response mechanisms and polarity-tunable characteristics provided by the 2D material platform can significantly enhance the extraction of target object features.
5. **Temporal Detector**: Temporal detectors can distinguish an object’s motion trajectory and state, typically storing each frame sequentially over time. As time is treated as a data dimension, the data volume is significantly larger and more complex compared to other dimensions. To reduce redundant data and backend processing time, advanced motion detectors encode temporal information as grayscale intensity, minimizing data redundancy (as shown in Fig. 4). In two-dimensional detectors, researchers have begun exploring event-driven detectors with even more compressed data volumes, greatly meeting the demand for real-time, low-power data processing.
6. **Phase Angle Detector**: The phase angle, as a critical wavefront parameter, is essential for describing the properties of light waves. Measuring parameters such as phase difference enables the acquisition of key information, including physical position and displacement, often identified through diffraction patterns or interference fringes. Two-dimensional material detectors leverage the material and structural topological characteristics to detect the OAM of vortex beams, thereby determining their helical phase distribution. These OAM states’ quantum numbers can be directly identified by the magnitude and polarity of the photoelectric response (as shown in Fig. 4).

### Overall strategy of multi-dimensional detectors

After introducing the six types of dimensional detectors and their parameter optimization methods, we are faced with a more technical challenge: how to integrate such a wide range of dimensional information into a single or multiple sub-pixel detector, while considering that some dimensional detections require multiple parameter inputs. Traditional scalar detectors, such as single-parameter intensity detectors, often lose complex input characteristics and end up producing distorted intensity information (lower panel of Fig. 4). The ultimate goal of multi-dimensional detectors is to design a single-pixel detector in 2D materials that can reverse the process and achieve information reconstruction (Fig. 4). Such a single multidimensional photodetector could be designed by employing spatial and temporal division strategies (Fig. 4). In the spatial division strategy, different dimensional detectors could be employed independently for each type, and then their various parameters could be fused and processed. In recent years, researchers have developed single-pixel, nonlinear electrically modulated multi-dimensional detectors. By utilizing a temporal division strategy, these devices have achieved breakthroughs in reducing device size. However, the underlying ‘black-box’ mechanisms remain unclear and often require neural networks for inverse calculation and assistance. On the other hand, vector photoelectric current multi-port devices, with minimal response crosstalk and stronger independence, are expected to solve the problem of temporal and spatial integration (Fig. 4).

## Classification of different types of new dimensional detectors

**Fig. 5:**
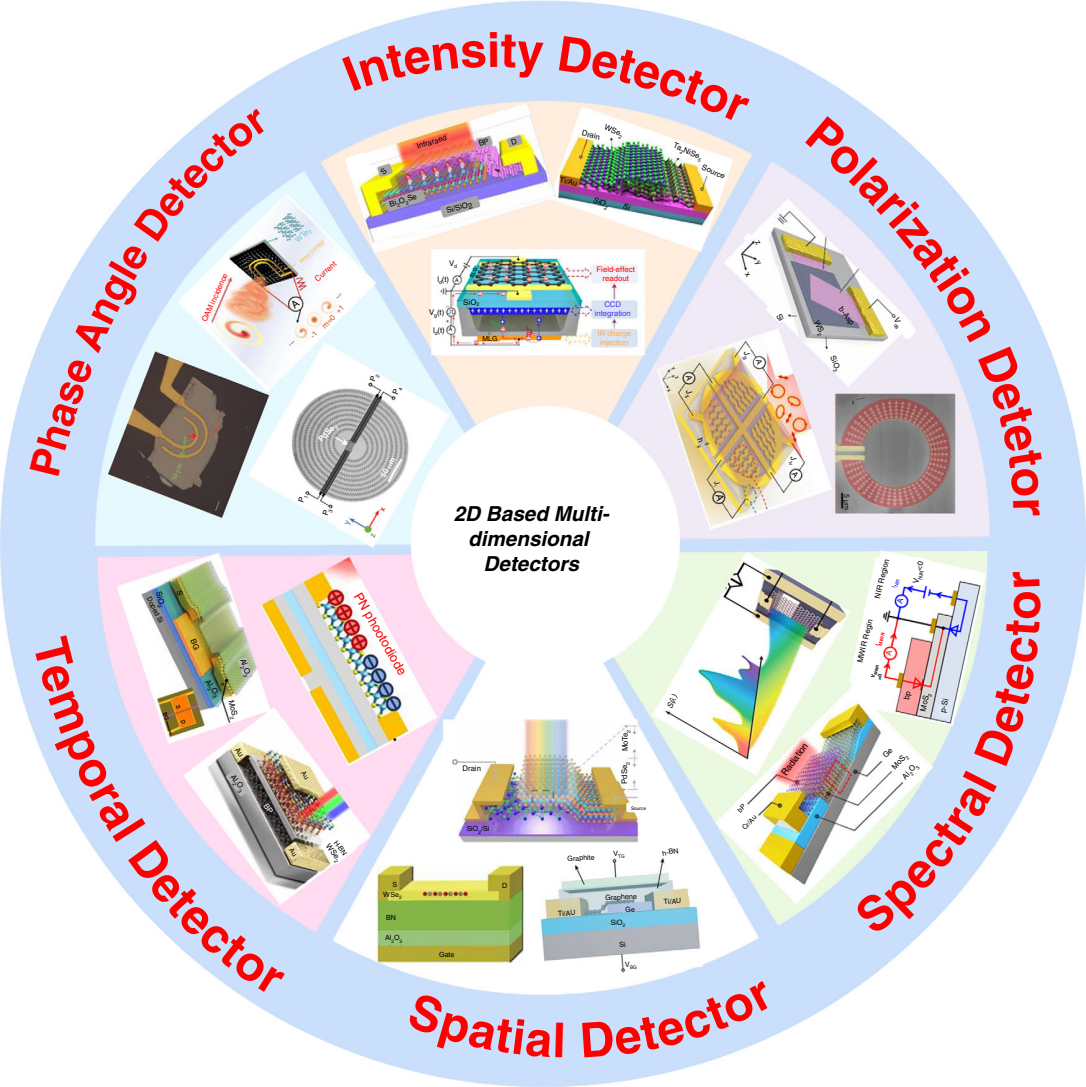
**Six types new dimensional photodetectors based on 2D materials**. Intensity detectors. Reproduced with permission^94,99,102^, Copyright 2022 and 2024, Nature Publishing Group; Copyright 2022, American Association for the Advancement of Science. Polarization detectors. Reproduced with permission^34,49,197^, Copyright 2022, Wiley-VCH; Copyright 2023 and 2024, Nature Publishing Group. Spectral detectors. Reproduced with permission^30,31,237^, Copyright 2022 and 2024, Nature Publishing Group; Copyright 2024, Optica Publishing Group. Spatial detectors. Reproduced with permission^136,138,139^, Copyright 2022 and 2024, Nature Publishing Group; Copyright 2020, American Association for the Advancement of Science. Temporal detector. Reproduced with permission^141,143,144^, Copyright 2022 and 2023, Nature Publishing Group. Phase angle detector. Reproduced with permission^52,53,207^, Copyright 2020 American Association for the Advancement of Science; Copyright 2022, Wiley-VCH; Copyright 2024, Nature Publishing Group

Before introducing multi-dimensional fusion detectors, we will provide a detailed overview of these six types of photoelectric detectors. Understanding the mechanisms and structures of existing novel dimensional detectors based on 2D materials will help us deeply analyze multi-dimensional detectors. These include intensity detectors, spatial detectors, temporal detectors, polarization detectors, phase angle detectors, and spectral detectors, as shown in Fig. 5. Each dimensional detector’s development will be introduced through structural or technological innovation pathways, with general design schemes also being discussed.

### Intensity detector

Intensity detectors, as the most basic and commonly used type of detectors, are widely applied in everyday life. Commercial detectors are typically categorized based on their power intensity detection range, such as photothermal, photonic, and multiplier types. By leveraging the defect-free interfaces, rich energy bands, miniaturization, high mobility, and excellent thermoelectric properties of 2D materials, intensity detectors with an ultra-wide linear dynamic range that surpass existing commercial detectors’ performance are expected to be realized through appropriate structural designs.

**Fig. 6:**
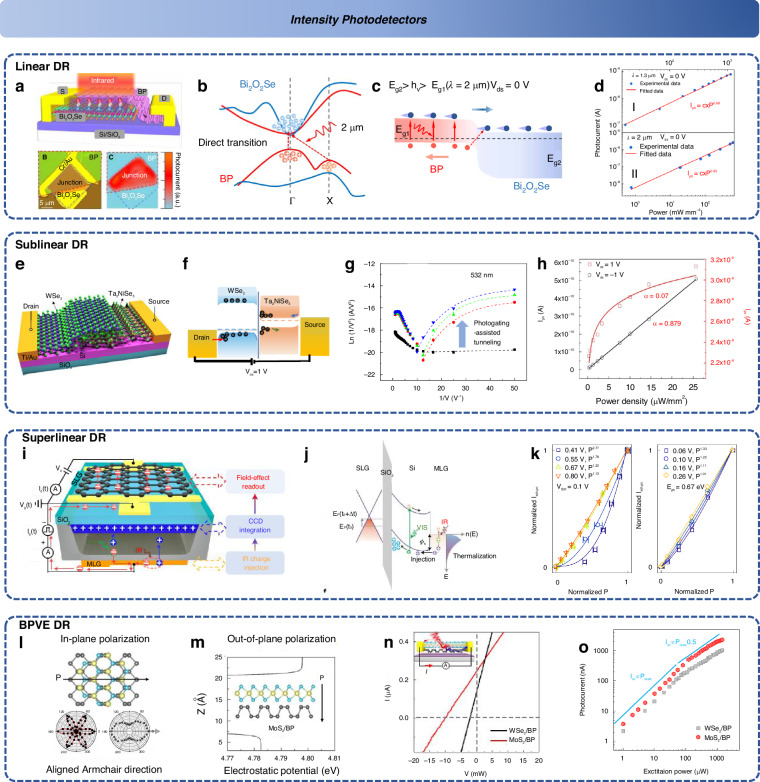
**Intensity detectors**. **a** The Van der Waals BP/Bi_2_O_2_Se framework^94^. **b** Energy matching interband transition^94^. **c** Heterojunction energy band of BP/Bi_2_O_2_Se^94^. **d** Linear relation in photocurrent-power plot^94^. Reproduced with permission^94^; Copyright 2022, American Association for the Advancement of Science. **e** The photogating WSe_2_/Ta_2_NiSe_5_ heterojunction device^99^. **f** The photogating energy band alignment^99^. **g** Photogating-assisted tunneling effect^99^. **h** The sublinear photocurrent-power trend^99^. Reproduced with permission^99^; Copyright 2024, Nature Publishing Group. **i** Graphene charge injection detector^102^. **j** The photothermionic emission effect in heterojunction^102^. **k** Superlinear response-power relation^102^. Reproduced with permission^102^; Copyright 2022, Nature Publishing Group. **l** The in-plane polarization of MoS_2_/BP^116^. **m** The out-of-plane polarization of MoS_2_/BP^116^. **n** The dual polarization BPVE versus single polarization BPVE in IV^116^. **o** The photocurrent-power relation in control devices^116^. Reproduced with permission^116^; Copyright 2024, Nature Publishing Group

In addition to sensitivity as a key parameter, the linear dynamic range is another crucial factor in evaluating the accuracy with which these intensity detectors restore input power, as power calibration is often unattainable in multi-dimensional detector architectures. Early research focused on constructing heterojunctions through PN junctions to achieve excellent photovoltaic and photonic detectors, such as Graphene/MoS_2_^86^, MoS_2_/WSe_2_^87^, MoS_2_/MoTe_2_^88^, and WSe_2_/MoSe_2_^89^, which demonstrated fast response speed and high responsivity. To further extend the detection wavelength, researchers explored more complex energy band alignments, proposing structures like the P-G-N sandwich (narrow-bandgap or semi-metal layers sandwiched between PN junctions)^90^, interlayer exciton transition^91,92^, NPs enhanced interlayer exciton^93^. And Chen et al. demonstrated BP/Bi_2_O_2_Se heterostructures (Fig. 6a) with momentum-matched interband transitions (Fig. 6b), which achieved higher efficiency and responsivity in the short-wavelength infrared range (Fig. 6c)^94^. Recently, more momentum-matched interband transitions are explored in 2D junctions^95–97^. These types of pure photovoltaic (PV) effect devices result in a purely linear (slope = 1) power-response relationship (Fig. 6d).

To further enhance detector response sensitivity, researchers have introduced gain mechanisms by employing the photogating effect, which leverages charge trapping to improve sensitivity^29^. Typically, photoconductive spatial charge trapping is utilized. In earlier studies, combining graphene, MoS_2_, and quantum dots achieved ultrahigh responsivity but at the cost of response speed^87,98^. Strategies based on interfacial state trapping in 2D material heterojunctions, as well as the intrinsic defect states on material surfaces, have utilized band bending at the junction interface to enable effective charge trapping, significantly improving responsivity. Recently, heterojunctions like WSe_2_/Ta_2_NiSe_5_ have demonstrated a novel photogating-assisted tunneling effect (Fig. 6e and g), where bias-controlled band structure changes regulate trap sites (Fig. 6f)^99^. This innovation breaks the traditional trade-off between responsivity and response speed. Additionally, researchers have used ferroelectric field modulation to control 2D photoconductive channels, achieving a robust photogating effect. Devices employing this mechanism exhibit sublinear relationships between photocurrent and response due to weak-light amplification effects as illustrated in Fig. 6h. Beyond planar gain mechanisms, researchers have also employed ballistic avalanche and in-plane avalanche mechanisms in two-dimensional materials, leading to a series of low-threshold, high-sensitivity devices capable of detecting extremely weak signals, such as those involving only tens of photons.

The aforementioned sublinear relationship is commonly observed in two-dimensional material heterojunction intensity detectors. These traps act as effective recombination centers, capturing charge carriers until high power levels cause saturation due to increased recombination, thus leading to sublinear behavior^83^. For superlinear response-power relationships, two typical mechanisms have emerged in two-dimensional materials. The first mechanism involves the emission of hot electron carriers that cross the interface barrier in graphene/hBN heterojunctions^100,101^. The photothermal effect induces an input that opens the Schottky barrier in a superlinear fashion, a phenomenon that has been reported in several studies^101^. This mechanism has been used to extend the wavelength range and enhance responsivity (as shown in the Fig. 6i)^102^. Using this mechanism, an infrared superlinear photodetector was realized with line array (Fig. 6j and k)^102^. Additionally, monolayers typically require engineered recombination centers (RC) for band engineering, usually possessing 2 to 3 states with varying density of states (DOS) parameters. At high power, the effective closing of RC centers is observed. This phenomenon has been discovered in multi-component alloy materials, such as MoS_2(1-x)_Se_2x_^103^, Ta_2_NiSe_5_^104^, and Ta_2_NiS_5_^105^, where the alloy defects result in multiple in-gap states.

To further break the Shockley-Queisser limit and achieve higher photovoltaic efficiency^106^, researchers have proposed utilizing 2D materials to disrupt the inherent symmetry in bulk crystals and realize the bulk photovoltaic effect (BPVE). This effect does not require junctions and only occurs in crystals where inversion symmetry is broken. This shift current phenomenon shows a transition from a linear dependence to a square root dependence^107^, a 2D based behavior observed in non-centrosymmetric, polarized multi-walled nanotubes made from curved two-dimensional transition metal dichalcogenides (TMDs)^108^. Subsequently, researchers have identified similar effects in various materials, such as WSe_2_/BP^109^, ReS_2_ homojunctions^110,111^, twisted bilayer graphene^37^, twisted bilayer BP^112^, 3R bilayer MoS_2_^113^, Strained 3R-MoS_2_^114^, and Te^115^. Recently, Zeng et al. demonstrated in MoS_2_/BP a unique in-plane/out-of-plane bipolar BPVE response (as shown in Fig. 6l and m)^116^. In comparison to the weaker out-of-plane response observed in WSe_2_/BP (Fig. 6n), MoS_2_/BP exhibited an overall enhanced response, with the photocurrent showing a stronger transition from linear dependence to square root dependence (Fig. 6o). Furthermore, the response speed reached 2.2 ns^116^. This shift current is associated with various physical mechanisms, including spin-lattice coupling and changes in the Bloch band orbital composition, making the linear dynamic range behavior more complex than previously observed^117,118^. This transition from linear dependence to square-root dependence shows potential for future applications in on-chip computation for adaptive image intensity detection, especially when such shift current transitions can be modulated electrically.

### Spatial detector

Spatial detectors typically require arrays for operation, which can determine the 3D position of objects by analyzing the intensity variations along different axes (front, back, left, right). To capture spatial images using arrays with millions of detector pixels, single-pixel cameras employ a fundamentally different approach: instead of a pixelated sensor array, they utilize a single-pixel detector combined with a series of mask patterns to spatially filter the scene^119,120^. The corresponding transmitted intensities are then recorded sequentially by the single-pixel detector. This indirect sampling and computational reconstruction imaging technique is generally applied in non-visible spectral bands^121^. Its underlying principle can be summarized into three key components: a high-sensitivity single-pixel detector, spatiotemporal modulation encoding, and compressive sensing reconstruction algorithms^122^. Typically, a single high-sensitivity detector (such as a photomultiplier tube or a superconducting nanowire single-photon detector) replaces the conventional pixel array in a camera, dedicated to capturing the total reflected or transmitted optical intensity from the scene. These detectors demonstrate advantages in low-light conditions or in specialized spectral regions, such as infrared or terahertz, making them particularly suitable for applications requiring high sensitivity or penetrative scene recognition^123,124^. Spatial light modulators (SLM) or digital micromirror devices (DMD) project a sequence of predefined encoding patterns (such as random binary matrices or Hadamard) bases-onto the object’s optical path, where each modulation corresponds to a distinct spatial structure. The intensity measurements acquired under these patterns enable subsequent computational reconstruction of the spatial image. The $k$-th modulation pattern ${{\rm{\phi }}}_{k}$ is projected onto the object, where the object’s reflected or transmitted light intensity distribution x is weighted by ${{\rm{\phi }}}_{k}$. The single-pixel detector then records the weighted total light intensity measurement ${{\rm{y}}}_{k}$: ${{\rm{y}}}_{k}=\mathop{\sum }\limits_{i=1}^{n}{{\rm{\phi }}}_{k}(i)\cdot x(i)$. In the temporal dimension, dynamic encoding is achieved by rapidly switching the modulation patterns, thereby converting the spatial information of the image into a temporal signal sequence^125^. Assuming the image is sparse in a specific transform domain, compressive sensing theory is employed to recover the original image from multiple linear projections obtained via sequential modulations. This is formulated as solving the underdetermined system ${\rm{y}}={\rm{\psi }}{\rm{\phi }}{\rm{s}}$ for ${\rm{s}}$ (${\rm{s}}$ represents the sparse coefficient vector) using optimization algorithms such as basis pursuit or iterative thresholding. Consequently, the original image can be reconstructed from a data volume significantly below the Nyquist sampling requirement^119^.

This technique substantially reduces hardware complexity and cost, with the system cost being approximately one-tenth that of traditional focal-plane array cameras^121^. It is particularly suitable for non-visible wavelength imaging, low-light environments, and high-speed dynamic scenes, while also reducing data acquisition volume and improving imaging efficiency. However, acquiring a single frame typically requires tens of milliseconds to several seconds (much slower than conventional cameras operating at frame rates above 30 fps) leading to potential motion artifacts. The reconstruction quality is highly sensitive to factors such as the choice of sparsifying basis, regularization parameters, and noise levels. When the image does not satisfy the sparsity assumption (e.g., scenes with complex textures) or when the measurement matrix and sparsifying basis are mismatched, reconstruction artifacts such as distortions and blurring may occur^126^. In addition to some existing studies that employ two-dimensional materials such as graphene for spatial detection^125^, the development of new material systems optimized for integration with spatiotemporal modulation encoding remains an open area of research and represents a promising future direction.

Moreover, 2D position detectors, particularly those based on 2D materials, have been extensively explored and are commonly used in applications such as laser beam alignment and stabilization^3^. These detectors operate based on the Schottky response and the lateral carrier movement determined by the geometrical center position of the light source. Electrodes positioned in two directions (x and y) can collect position information across the entire plane. In this context, Lu et al. designed a series of detectors based on graphene/Si^127^, graphene/Ge^128^, and graphene/p-Si/n-Si heterostructures^129^, with response speeds in the µs range. These detectors achieved a spatial resolution of 1 µm and a minimum detectable power in the nW range, and they have already been applied to multi-target trajectory tracking.

Additionally, we mention another class of spatial detectors, which function more as spatial response processors. Traditionally, spatial position differences have been resolved using array-based and curved surface detectors. However, 2D material-based spatial detectors primarily focus on how to operate using linear and nonlinear by Hadamard products^83,130^. The Hadamard product is defined as the element-wise multiplication of two matrices. In the context of visual detection, this operation can be applied between a visual spatial detector and a visual spatial scene (considering 2D spatial scene), with their relationship expressed by the following formulation^130^:

$$\left(\begin{array}{ccc}{R}_{11} & \cdots & {R}_{1n}\\ \vdots & \ddots & \vdots \\ {R}_{m1} & \cdots & {R}_{{mn}}\end{array}\right)\odot \left(\begin{array}{ccc}{W}_{11} & \cdots & {W}_{1n}\\ \vdots & \ddots & \vdots \\ {W}_{m1} & \cdots & {W}_{{mn}}\end{array}\right)=\left(\begin{array}{ccc}{O}_{11} & \cdots & {O}_{1n}\\ \vdots & \ddots & \vdots \\ {O}_{m1} & \cdots & {O}_{{mn}}\end{array}\right)$$

Where the R represents the spatial distribution of response to real-world information, W represents the response feature weights of the visual detector. Through this operation, spatial response information can be selectively enhanced or extracted. The matrix dimensions (m${{\times }}$ n) are intrinsically linked to the system’s resolution. Furthermore, by adjusting the characteristics of the visual detector, various mapping operations can be performed on the input information and output features, enabling flexible feature extraction and transformation.

**Fig. 7:**
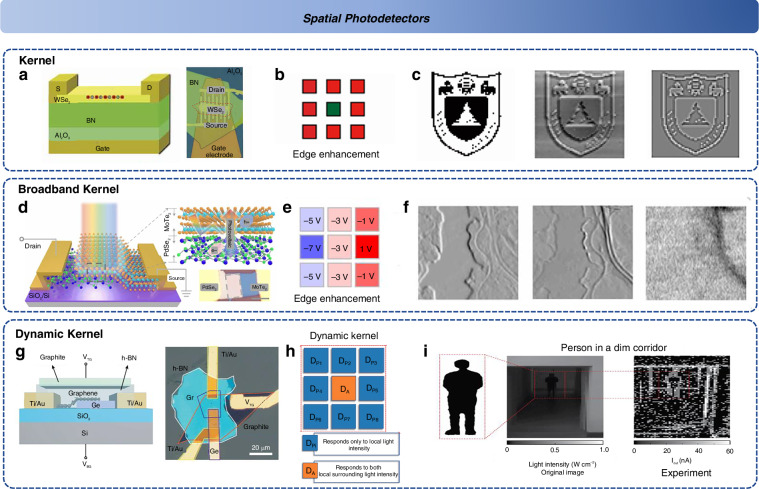
**Spatial detectors**. **a** The retinomorphic device based on WSe_2_/h-BN^136^. **b** The Laplace operation in image convolution process^136^. **c** The edge enhancement results (origin, experiment, simulation)^136^. The Reproduced with permission^136^; Copyright 2020, American Association for the Advancement of Science. **d** The device structure and image of PdSe_2_/MoTe_2_ heterojunction^138^. **e** The Sober edge enhancement of convolution process^138^. **f** The broadband edge enhancement of topographic map (UV, VIS, NIR)^138^. Reproduced with permission^138^; Copyright 2022, Nature Publishing Group. **g** The framework and optical image of the graphene/Ge device^139^. **h** The dynamic kernel of convolution process^139^. **i** Edge detection and recognition in dim lighting condition^139^. Reproduced with permission^139^; Copyright 2024, Nature Publishing Group

This type of spatial data preprocessing helps alleviate the precision issues in spatial information recognition that are typically encountered with traditional detectors, enhancing the processing of useful, feature-rich data within a given spatial context^130^. In 2019, Zhou et al. achieved noise suppression in spatial images via a time-dependent nonlinear response, enhancing the signal-to-noise ratio and emphasizing the characteristic signals in the space^131^. However, this approach requires time accumulation, demanding significant bandwidth. To reduce intermediate processing times, Mennel et al. proposed, for the first time, the use of gated-reconfigurable bipolar arrays made from few-layer WSe_2_ to achieve on-chip perception and processing of optical images^132^. These image sensors themselves function as an artificial neural network (ANN), accomplishing tasks such as classification, encoding, and denoising. The recognition response speed was demonstrated to be as fast as 50 ns, underscoring the potential of 2D materials for constructing more complex spatial response processors^132^. These gated-reconfigurable devices can also simulate more biologically relevant neural imaging systems, such as the ON and OFF cells in bipolar cells of the cone-rod system, thereby realizing the visual preprocessing process^46,133–135^. In 2020, Wang et al. developed a WSe_2_/h-BN/Al_2_O_3_ structure (Fig. 7a) that utilized defect-induced electron carrier transport in h-BN to achieve a bipolar optical response, with a response speed of 8 ms^136^. Furthermore, leveraging this effect, they built a reconfigurable retinal visual sensor capable of achieving image stylization, edge enhancement, and contrast enhancement (Fig. 7b and c) by employing the Laplacian operator, which is more analogous to the human visual system^136^. In 2021, the same group combined the above device with a memristor, thereby achieving image sensing, processing, and recognition^137^. This setup mimicked the entire framework from the visual system to the visual cortex, demonstrating that processing spatial information is indeed feasible. These works all relied on the Laplacian operator for edge enhancement, though it is susceptible to noise interference. In 2022, Pi et al. used the gate control of PdSe_2_/MoTe_2_ heterojunctions to induce an internal built-in electric field reversal (Fig. 7d)^138^. This bipolar photovoltaic response was achieved across a wide ultraviolet to near-infrared wavelength range. With good photovoltaic response linearity, the Sober operator was used to enhance the edge image (Fig. 7e). The Sober operator offers better noise suppression, and this wideband convolutional processing allows for the extraction of more spatial features from remote sensing scenes (Fig. 7f)^138^. In low-light conditions, where the signal-to-noise ratio is lower, static edge enhancement operators are insufficient. In 2024, Yang et al. constructed a 3 × 3 device array based on Graphene/Ge (Fig. 7g), and for the first time, employed dynamic convolution kernels (Fig. 7h)^139^. The active components within this array modulate their response based on the photocurrent output from adjacent pixels and the local image gradient, thus enabling the extraction and effective recognition of dim targets (Fig. 7i)^139^.

2D material-based spatial response processors require large-area arrays and must account for array consistency effects and the complexities of differential circuits. Additionally, compatibility with traditional CMOS technologies is necessary for future on-chip integration and practical deployment. In the future, spatial response processing within sensors is expected to extend into the mid-infrared wavelength range.

### Temporal detector

Traditional CMOS imaging cameras used for temporal (motion) detection have fixed frame rates, recording complete image information for each frame. This results in the collection of a large amount of redundant data, while capturing the truly useful (dynamic) information becomes difficult. This imposes a significant energy consumption burden on backend processing units. However, with recent advances in backend graphics processing unit (GPU) developed by companies like NVIDIA, the development of front-end motion image detectors seems to have become less urgent. Nevertheless, the issue of data redundancy in traditional motion detection remains unresolved. When combined with existing high-performance processors, especially in applications where useful information needs to be extracted from complex environmental monitoring, there is potential for the creation of more powerful motion detection camera systems. Spatiotemporal detectors share many connections with spatial detectors. Given the limited bandwidth of detectors, the way in which tasks are allocated must be studied according to specific circumstances. For example, in human motion detection, parallel and serial processes can be considered: the direction perception of short-distance movements can be processed in parallel, while more complex long-distance movements may require combined processing, involving more detectors^140^. Therefore, the primary goal of motion detectors today is to control motion data acquisition based on amplitude-domain signal changes, adjusting the acquisition frequency according to dynamic scene requirements, ultimately enabling the detection of the full physical dynamics (velocity extraction, motion anticipation, and motion extrapolation) of motion^130^.

To effectively reduce hardware resources, detectors based on two-dimensional materials with memory capabilities can be used to process dynamic information. Early research achieved the encoding of input optical information in the time domain using spike-intensity photoelectric current data and memory-modulating devices. Synaptic-like devices with bound states have advanced to the point where they can encode input optical information over time, although they were initially limited to static image recognition. However, these works have provided partial inspiration for realizing time-dependent motion detectors.

**Fig. 8:**
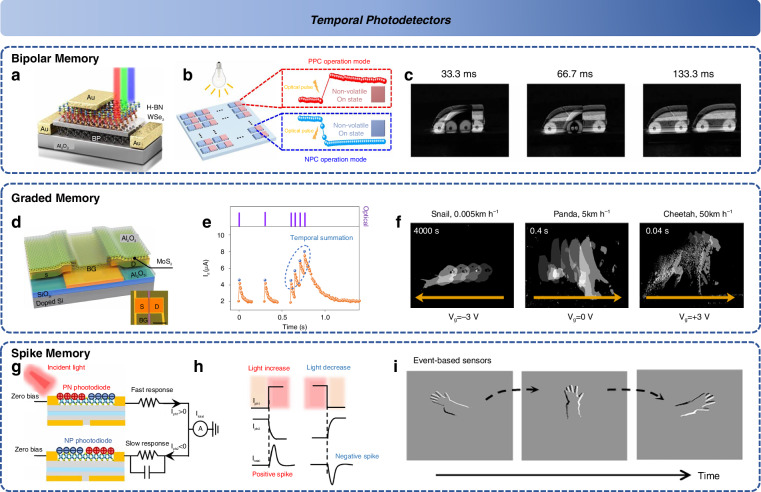
**Temporal detectors**. **a** Bipolar memory by floating gate BP/h-BN/WSe_2_ structure^141^. **b** Retina-inspired NPC and PPC modes^141^. **c** Motion detection result with various time interval^141^. Reproduced with permission^141^; Copyright 2022, Nature Publishing Group. **d** The MoS_2_ phototransistor^143^. **e** Light pulse-induced time-resolved photocurrents^143^. **f** Image detection at different motion speeds^143^. Reproduced with permission^143^; Copyright 2023, Nature Publishing Group. **g** The two parallel and programmable WSe_2_ detectors with different capacitances^144^. **h** Output spikes with light increase and decrease^144^. **i** Event driven for motion image^144^. Reproduced with permission^144^; Copyright 2023, Nature Publishing Group

From the early stages of processing response through time-domain intensity output, motion detection has gradually matured into simple image capture for specific motion systems. In 2022, Zhang et al. developed a motion detector using a BP/h-BN/WSe_2_ memory floating-gate device architecture (as shown in Fig. 8a)^141^. This device enables the positive and negative memory of each frame (Fig. 8b), where adding the recorded positive and negative image results retains only the motion output (Fig. 8c). This significantly reduces the transmission and interaction of extraneous information. Additionally, this work can simulate the visual receptive field, extracting edge feature information, thereby preliminarily demonstrating some fundamental characteristics of spatiotemporal image detectors^130,141^. Furthermore, Pang et al. utilized undulated MoS_2_ devices to construct an 18 × 18 photoelectric array capable of achieving bipolar photo-memory across a wavelength range of 405-940 nm^142^. This device demonstrated a dynamic light storage range exceeding 10^6^, making it suitable for motion detection and recognition in both bright and dark environments^142^.

However, the bipolar memory device still has limitations in detecting motion direction, which could impact subsequent motion prediction and extrapolation. Considering this, in 2023, inspired by biological graded neurons, Chen et al. employed MoS_2_ devices with shallowly bound traps (Fig. 8d) to simulate encoding with temporal frame rate information^143^. This graded response further enhanced the data transmission rate, reaching up to 1200 bit/s (Fig. 8e), and improved the trajectory recognition of moving targets (Fig. 8f)^143^. To reduce redundant signals, Zhou et al., in the same research group, constructed a motion detector by integrating two WSe_2_-based homojunctions with opposite polarities in parallel (Fig. 8g)^144^. This configuration allowed the detected data to be directly encoded into spike signals, which were subsequently processed by a digital neuromorphic processor (Fig. 8h), eliminating the need for time and energy-consuming transmission conversions. The device registered positive spikes for increasing light intensity and negative spikes for decreasing intensity, which was ultimately reflected in the motion signal detection of hand gestures, allowing for the determination of gesture direction based on positive and negative images (Fig. 8i)^144^. In 2024, Wu et al. further optimized this structure by combining the pulsed photoelectric detector with artificial synapses, compensating for lost information and continuing to reduce energy consumption^145^.

Currently, the motion detectors implemented in the array here do not belong to the same category as the aforementioned spatial detectors. The relevant devices do not perform position recognition or spatial information processing; therefore, this section only involves the time encoding process after the spatial data has been determined. Furthermore, the above-mentioned motion detectors are not frame-rate adaptive (i.e., the output does not self-adjust based on the magnitude of motion). Instead, the output remains a uniformly adjusted frame rate after modulation. This type of passive adjustment device still has limitations in detecting non-uniform or curved spatial motion objects, falling short of realizing a true spatiotemporal two-dimensional image detector.

### Polarization detector

Polarization detectors, which are capable of discerning the oscillation direction and characteristics of light waves, have found extensive applications in both civilian and military domains^4^. In industrial settings, polarization cameras play a critical role in non-destructive testing, pharmaceutical quality control, and structural integrity assessment of construction materials^146,147^. Additionally, polarization imaging effectively mitigates glare interference, thereby enhancing the accuracy of traffic violation monitoring systems^148^. In the field of communications, polarization-based techniques have demonstrated promising preliminary applications, particularly in anti-jamming communications and quantum encryption^149^. Meanwhile, in biomedical imaging, polarization detection leverages the optical anisotropy of biological tissues to significantly improve the sensitivity of early cancer diagnosis^150^. Currently, polarization technology is undergoing rapid advancements through interdisciplinary innovations, driving progress in diverse fields such as autonomous driving (polarization-based dehazing), remote sensing, and environmental pollution monitoring^151,152^. These developments underscore its immense technological potential and broad applicability.

Polarization is a complex vector parameter that typically exists in three states: linear polarization, circular polarization, and elliptical polarization, and is often described using the Poincaré sphere to represent Stokes parameters^153^. Traditional optical polarization detectors employ strategies such as temporal division, amplitude division, and focal plane division. However, these detectors generally suffer from drawbacks, including complex architectures, large size, and slow response speed. A notable example of a commercial device is the Sony four-pixel polarization detector, which features four different wire-grid directions^154^. This device has been applied in areas such as stress detection and surface reflection reduction.

In recent years, advancements in 2D materials have shown promise in overcoming the limitations of traditional polarization detection technologies^4,155,156^. By leveraging material lattice structures, energy band differences, and physical states, ultra-compact full-Stokes polarization detectors may become achievable^157^. Based on specific applications and measurement methods, 2D material structures have progressed from simple linear and circular polarization detection to angle-resolved linear polarization detectors with two-parameter inputs, and ultimately to detectors capable of measuring full-Stokes polarization^154^. Early studies utilized the intrinsic anisotropy of 2D materials to achieve clear linear polarization detection^158^. Extensive research has been conducted on orthorhombic, triclinic, and monoclinic crystal systems. Among these, the orthorhombic system contains a variety of notable materials, such as BP^159^, Asp^160^, PdSe_2_^161^, GeSe_2_^162^, Ta_2_NiSe_5_^163^, and Ta_2_NiS_5_^164^. Other crystal systems, including ReS_2_^165^, ReSe_2_^166^, and GeS_2_^167^, have also gained significant attention. Visible-light polarization has been explored in ReS_2_^165^, and ReSe_2_^166^, with heterojunctions like WSe_2_/ReSe_2_^168^, ReS_2_/In_2_Se_3_^169^, and ReS_2_/SnSe_2_^75^ achieving polarization ratios (PR) of 1.5-2.8 in the visible range.

Quasi-one-dimensional crystals such as Ta_2_NiSe_5_ and Ta_2_NiS_5_ exhibit stronger infrared polarization capabilities^163,170^. Recently, studies on exciton insulators have been reported, including Ta_2_NiSe_5_/WSe_2_^171^, Ta_2_NiSe_5_/MoTe_2_^172^, Ta_2_NiS_5_/BP^72^ P-N junctions, which have achieved near-infrared PRs of up to 1.44. For the mid-infrared range, researchers have utilized narrow-bandgap 2D materials to construct heterojunctions such as BP/MoS_2_^173^, Asp/MoS_2_^174^, Asp/MoTe_2_^175^, BP/InSe^176^, BP/PVDF^177^, PdSe_2_/MoS_2_^178^, and PdSe_2_/Graphene^69^, achieving PR coverage from 1 to 10.

Recently developed ternary alloy materials, such as NbIrTe_4_^179^, TaIrTe_4_^180^, Nb_2_GeTe_4_^181^, and Nb_2_SiTe_4_^182^, have extended linear polarization detection wavelengths to 10.6 μm, with single-material PR reaching 1.88. Multilayer structures appear to exhibit stronger linear PR: fully depleted junction WSe_2_/Ta_2_NiSe_5_/WSe_2_ (PR = 14.8)^183^, MoTe_2_/Ta₂NiSe_5_/MoTe_2_ (PR = 16.3)^184^, MoSe_2_/GeSe/MoSe_2_ (PR = 12.5)^185^, back-to-back heterojunctions BP/MoS_2_/BP (PR = 100)^186^, and Graphene/PdSe_2_/Ge (PR = 112.2)^187^. Additionally, unipolar barrier strategies such as BP/MoS_2_/Graphene (PR = 4.9)^27^, BP/MoS_2_/Asp (PR = 35.5)^78^, and encapsulation strategies like hBN/Asp/hBN (PR = 14)^188^ have also been developed.

**Fig. 9:**
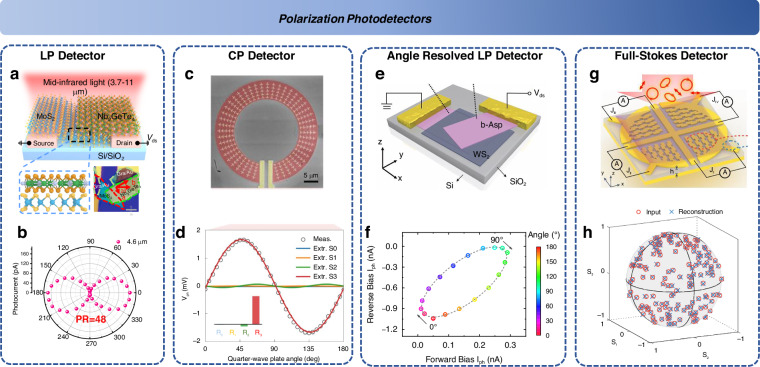
**Polarization detector**. **a** Anisotropic Nb_2_GeTe_4_ based LP detector^73^. **b** Polar plots of LP photocurrents^73^. Reproduced with permission^73^; Copyright 2023, Wiley-VCH. **c** The structure of Graphene/ring-distributed centrosymmetric metasurface^34^. **d** The angle dependent CP result^34^. Reproduced with permission^34^; Copyright 2023, Nature Publishing Group. **e** The b-Asp/WS_2_/b-Asp back to back detector^49^. **f** 2D plots of bias dependent output with different LP states^49^. Reproduced with permission^49^; Copyright 2022, Wiley-VCH. **g** Full Stokes detector with MoS_2_/four-subpixel metasurface framework^197^. **h** Reconstructed polarization states in Poincaré sphere^197^. Reproduced with permission^197^; Copyright 2024, Nature Publishing Group

In addition, the integration of two-dimensional materials with plasmonic structures has enabled the engineered design and optimization of polarization detectors. Li et al. demonstrated continuous tunability of the PR from 0.2 to 1 by combining MoTe_2_ with a metasurface^189^. Similarly, Yu et al. achieved high PR (2.5-4.6) performance in the narrow-band near-infrared by integrating graphene with a dielectric distributed Bragg reflector^190^. In 2020-2021, Wei et al. first proposed a strategy to achieve extreme PR^51,58^. By constructing heterogeneous metasurfaces on graphene, they successfully developed a reconfigurable approach capable of fully realizing transitions from 1 → +∞ and -∞ → -1^58^. In the pure van der Waals domain, In 2024, Wang et al. utilized gate-tunable flow characteristic inversion in 1T’-MoTe_2_/WSe_2_ to achieve a similar reconfigurable strategy in the visible wavelength range^191^. Han et al. using the P-N junction of Nb_2_GeTe_4_/MoS_2_ (Fig. 9a), achieved reversed photothermal responses, enabling bipolar polarization detection at 3.7 μm with a maximum mid-infrared PR of 48 (Fig. 9b)^73^.

In addition to linear polarization, circular polarization has also been extensively studied in 2D materials. In 2023, Gu et al. achieved a circular polarization g-factor of 0.5 at a wavelength of 405 nm using 2D covalent organic frameworks (COFs)^192^. However, materials with intrinsic chirality are rare in two-dimensional systems. Therefore, circular polarization detection is often realized by breaking in-plane and out-of-plane symmetries or combining chiral metasurfaces with 2D materials. Che et al. constructed a PdSe_2_/2H-MoTe_2_ heterojunction and leveraged the hidden spin polarization and inversion spin Hall effect in 2H-MoTe_2_ to achieve circular polarization detection at 1310 nm in the near-infrared range^80^. Researchers have also discovered circular photocurrent effects (CPGE) in TMDs, topological insulators, Weyl semimetals, and Weyl semiconductors^193^. For instance, circularly polarized oblique incidence on monolayer or few-layer MoS_2_ can break in-plane and out-of-plane symmetries, resulting in valley-associated inequivalent excitations and enabling circular polarization discrimination^194^. Additionally, due to the rich helical-dependent optical selection rules within and between Weyl cones in Te, wavelength-dependent circular polarization responses can be observed^79^. However, circular polarization detectors often exhibit linear polarization redundancy components, which can affect circular polarization detection accuracy. Wei et al. addressed this by employing mirror-symmetric metasurfaces arranged in a circular pattern (Fig. 9c), achieving a circular polarization g-factor as high as 84 and suppressing non-circular polarization field components (Fig. 9d)^34^. Recently, He et al. developed a pure circularly polarized light detector by integrating a gradient metasurface with InSe, effectively suppressing the response to non-circularly polarized light^195^.

The aforementioned works focus on linear or circular polarization detectors. However, linear polarization detectors lack a parameter to uniquely determine the linear polarization angle, as the photocurrent exhibits a single turnover with respect to the polarization angle. To address this, two linear polarization detectors with different initial phase angles are needed. Deng et al. constructed a back-to-back Asp/WS_2_/Asp structure with a 70° angle between the two Asp layers (Fig. 9e)^49^. This configuration allows two polarization detectors with different phase angles to determine the input linear polarization angle (Fig. 9f), classifying this work as a time-division polarization detector^49^. Elliptical polarization detection, requiring more degrees of freedom, demands full-Stokes detectors. Full-Stokes detectors have been explored in metasurfaces, where designing appropriate metasurface structures is key to achieving such detection. Additionally, metasurfaces can optimize transmission matrices, thereby enhancing reconstruction accuracy. Dai et al. utilized PdSe_2_ combined with chiral metasurfaces to generate PTE, constructing an on-chip configuration for mid-infrared full-Stokes detection with PR = -1 and g-factor of ∞, achieving three-port functionality^196^. In 2024, Deng et al. proposed a compact four-pixel on-chip full-Stokes detector (Fig. 9g)^197^. They introduced the concept of photoelectric polarization eigenvectors and clarified the linear relationship between Stokes vectors and photocurrent. Using Gaussian process regression, they achieved an azimuthal angle accuracy of 0.69° and an ellipticity angle accuracy of 0.51° (Fig. 9h)^197^.

### Phase angle detector

Phase information, as a crucial characteristic of the wavefront, can be lost or disturbed during propagation due to non-vectorial superposition effects. In traditional optics, extensive research has been conducted on capturing the absolute phase difference of waves using phase-locked loops or interferometers. However, this paradigm has been scarcely explored in the context of 2D material-based optoelectronic detectors. However, photodetectors based on 2D materials often lack the “phase-sensitive volume” or interference conditions required to detect continuous phase variations, making it difficult for them to replace conventional technologies. In contrast, the discrete nature of OAM modes makes them more easily detectable through photocurrent or photothermal responses in 2D materials. Moreover, certain 2D materials exhibit strong coupling responses to polarization and OAM (such as in valleytronics) and demonstrate selective absorption of OAM during interband transitions. In contrast, OAM detectors have been the subject of in-depth investigation within 2D material-based detection systems.

Vortex beams, whose wavefront phase exhibits a helical structure, are capable of carrying both spin angular momentum (SAM) and OAM^198^. With their exceptional robustness against interference, vortex beams have been widely utilized in optical communication, target detection, and quantum information processing^199,200^. The relationship between SAM and OAM can be analogized to the motion of electrons around a nucleus: the angular momentum generated by the orbital motion of an electron corresponds to OAM, while the momentum arising from the electron’s spin corresponds to SAM^201^. Traditional optical OAM detection methods typically employ holographic gratings to convert OAM-mode beams into planar Gaussian beams, enabling the detection of individual OAM modes^202^. Additionally, interferometric techniques, such as annular aperture interference and Mach-Zehnder interferometers, are used to detect optical OAM by analyzing the interference patterns of two beams with opposite OAM modes and determining the topological charge based on the number of fringes^203–205^. However, these conventional methods face limitations due to the complexity of optical path design, manufacturing precision constraints, and resolution issues. Moreover, they often rely on indirect detection approaches, restricting their practical applicability. To address the demands of next-generation compact devices, novel direct OAM detection technologies have been developed, offering promising advancements in this field.

**Fig. 10:**
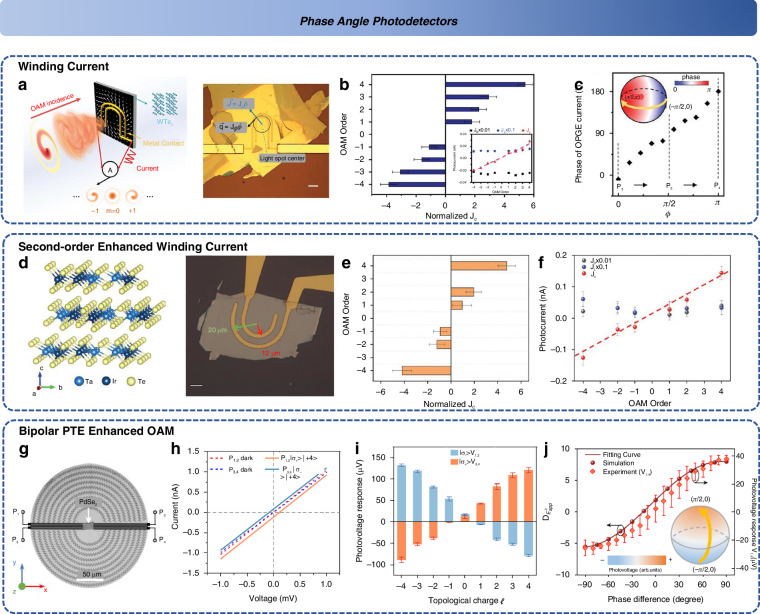
**Phase angle detectors**. **a** The WTe_2_ integrated with U-shaped electrode^52^. **b** The output photocurrent based on OPGE^52^. **c** The phase of OPGE current^52^. Reproduced with permission^52^; Copyright 2020, American Association for the Advancement of Science. **d** The Weyl semimetal TaIrTe_4_ coupled with U-shaped electrode^53^. **e** OAM discrimination as function of J_c_^53^. **f** OAM correlation of different component currents^53^. Reproduced with permission^53^; Copyright 2022, Wiley-VCH. **g** The spin Hall coupler integrated with PdSe_2_^207^. **h** Bipolar PTE response with different OAM states^207^. **i** OAM ellipticity discrimination^207^. **j** Phase difference as the function of simulation and experiment^207^. Reproduced with permission^207^; Copyright 2024, Nature Publishing Group

The emergence of 2D materials provides opportunities to further optimize and enhance the extraction of OAM (orbital angular momentum) beam information by exploring the photogalvanic effect and symmetry selection rules of 2D materials^54^. Specifically, materials with broken inversion symmetry, such as C_2V_ point group type II Weyl semimetals, can suppress associated photocurrents^52^. Under circular photogalvanic effects (CPGE), the symmetry in materials like WTe_2_ also prohibits the generation of photocurrents under linearly polarized light. Moreover, the photon drag effect is also prohibited under normal incidence. Ultimately, only information-carrying beams with OAM (spatially dispersive photogalvanic effect and orbital photogalvanic effect) are symmetrically allowed^52^. Current vortices collected by U-shaped electrodes can distinguish spiral phase gradients. Subsequently, Lai et al. demonstrated, using the mid-infrared C_2V_ point group Weyl semimetal TaIrTe_4_ with U-shaped electrodes (Fig. 10d), that topologically enhanced responses via shift currents lead to a linear dependence of the CPGE component current on the OAM order (Fig. 10e)^53^. Furthermore, the anisotropy and polarization components exhibit a dependence on the OAM order of the non-surface states (Fig. 10f). This enables on-chip direct detection in the mid-infrared wavelength range.

With the development of metasurfaces, the detection of OAM (orbital angular momentum) light using plasmonics (surface plasmon polaritons, SPP) has become a promising approach. In 2016, Mei et al. proposed using semi-ring plasmonic nano-slits to spatially classify OAM light by focusing SPPs, thus enabling the identification of vortex light with different topological charges^206^. To further reduce the spatial footprint and achieve compact detection, Ren et al. multiplexed the nano-ring slits, achieving a low mode crosstalk of -17 dB, and demonstrated a multi-mode sorting with a spatial footprint of 4.2 μm × 4.2 μm^206^. However, this approach still requires far-field detectors and microscopes to read the relevant information. To enable on-chip devices, in 2024, Dai et al. integrated photothermoelectric 2D materials (PdSe_2_) with spin-Hall SPP structures (Fig. 10g)^207^. This integration transformed previous spatial distribution detection into photovoltage amplitude and polarity measurements (Fig. 10h). The photovoltage response is primarily determined by the location of the SPP hotspot, exhibiting good mode resolution (Fig. 10i). Additionally, the bipolar photovoltage mechanism enhances the mode resolution of vortex light. Using this polarity response, it is possible to distinguish the chirality and ellipticity of fixed-mode OAM scalar vortex beams (Fig. 10j)^207^. Recently, Yang et al. demonstrated that even topologically trivial graphene can be used for OAM detection, providing a promising device platform for future on-chip integration^208,209^.

### Spectral detector

Single-spectrum detectors often lose spectral information, leading to incomplete data acquisition^210^. Multi-spectral and spectrally tunable detectors, which enable switching between visible, near-infrared, and long-wave infrared, can construct comprehensive visual information and improve target recognition accuracy^211^. These tunable detectors have extensive applications in fields such as space exploration, defense, agriculture, and biology^212–214^. Furthermore, multifunctional bio-detectors based on advanced wavelength-selective materials can be employed for high-resolution and high-sensitivity genetic screening, demonstrating significant potential for early epidemic diagnosis and rapid disease differentiation^215–218^. For example, Earth observation satellites require multi-band spectral capture of surface reflections to extract material composition information, while dual-band infrared imaging in military reconnaissance can penetrate smoke and camouflage. 2D materials, with their broad spectral range and high electro-tunability compared to traditional materials, show promise for developing compact and highly adjustable spectral detectors. In the future, integrating artificial intelligence is expected to enable automated analysis and real-time processing of spectral detection data. Early approaches to multi-spectral detection involved stacking separate spectral materials and utilizing broadband or selective photodetectors^219^. These typically required complex fabrication and epitaxial processes, such as HgCdTe dual-band detectors^220^, and quantum dots (QDs) dual-band detectors^221^, which often suffered from significant inter-band crosstalk. Sub-four-pixel detectors with four-color capabilities exhibit less crosstalk but have larger pixel sizes^222^. Additionally, traditional materials require low-temperature cooling systems and epitaxial growth processes. Recently, Koepfli et al. demonstrated that graphene combined with metasurface structures can achieve broadband spectral coverage from 1400 to 4200 nm while maintaining a bandwidth of 500 GHz^223^. This work suggests that a broader range of two-dimensional materials and metastructures holds promise for future applications in computational spectral resolution.

**Fig. 11:**
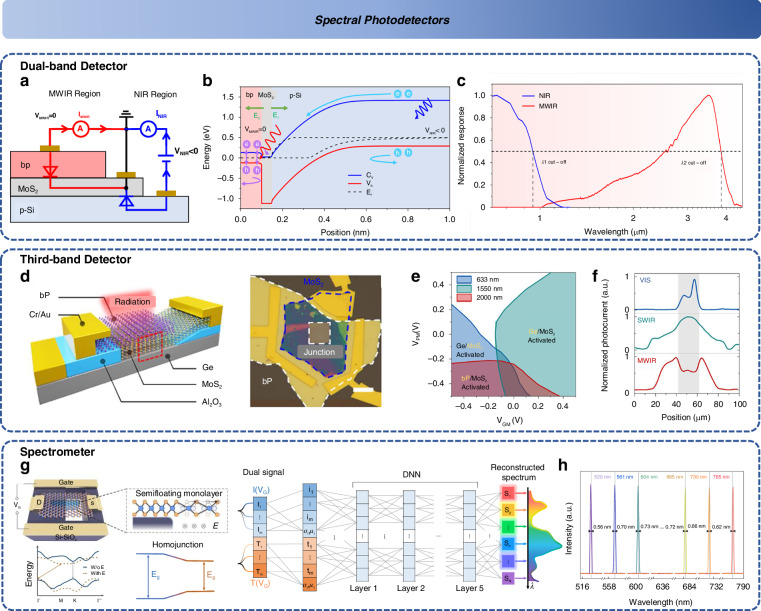
**Spectral detectors**. **a** Dual-band detector based on BP/MoS_2_/p-Si^31^. **b** Back to back energy band alignment^31^. **c** The response spectrum of BP/MoS_2_ and MoS_2_/p-Si^31^. Reproduced with permission^31^; Copyright 2022, Nature Publishing Group. **d** The third-band detector of BP/MoS_2_/Ge^30^. **e** Corresponding photocurrent distribution of BP/MoS_2_/Ge^30^. **f** The response spectrums of third-band modes^30^. Reproduced with permission^30^; Copyright 2024, Optica Publishing Group. **g** MoS_2_ homojunction spectrometer by strain engineering to tuning the band structure with input dual signal to reconstruct spectrum^237^. **h** Spectral resolution of spectrometer^237^. Reproduced with permission^237^; Copyright 2024, Nature Publishing Group

With extensive research into spectral responses, 2D materials have identified numerous high-performance van der Waals heterojunctions for specific wavelength ranges, such as MoS_2_/WSe_2_ for visible light^87^, MoS_2_/MoTe_2_ for near-infrared^92^, and MoS_2_/BP for mid-infrared detection^186^. To enhance CMOS compatibility, researchers have combined 2D materials with Si, Ge, and QDs to develop innovative dual-band detectors. In 2022, Chang et al. developed a fully 2D back-to-back near-infrared to mid-infrared dual-band detector based on a BP/MoS_2_/MoTe_2_ structure^224^. It required positive and negative bias modulation and achieved an optimized response time of 41 ns. The same year, Wu et al. fabricated a mixed-dimensional BP/MoS_2_/p-Si dual-band detector (Fig. 11a)^31^. Under positive and negative bias, BP/MoS_2_ and MoS_2_/p-Si operated independently, with the depletion region effectively blocked by the intermediate layer (Fig. 11b), achieving ultra-low inter-band crosstalk of 0.05% in the infrared to mid-infrared range (Fig. 11c)^31^. To further simplify material stacking and operation, Wang et al. spin-coated PbS QDs onto BP/MoS_2_, enabling near-infrared to mid-infrared dual-band detection under zero or forward bias, significantly reducing energy consumption compared to traditional bipolar bias structures^225^. Furthermore, the band-to-band tunneling (BTBT) mechanism is employed for spectral control, particularly in narrow-bandgap/wide-bandgap heterostructures. By tuning the applied bias, the tunneling process can be controlled, allowing for spectral response modulation via selective material activation. Hwang et al. developed a simple MoS_2_/Ge mixed-dimensional heterojunction that utilizes a BTBT, enabling visible to near-infrared dual-band detection without requiring extra voltage polarity switching^226^.

To overcome the limitations of dual-band detection, two types of multi-color detectors have been explored. In 2024, Shen et al. utilized a type-I junction BP/MoTe_2_, where gate voltage control of band alignment allows for nearly independent contributions from both materials^227^. This enables four spectral characteristics to be adjusted, including mixed bands, wide frequency bands, MWIR/SWIR, and NIR/visible bands, with an inter-band crosstalk of approximately 5%. Additionally, Wang et al. constructed a BP/MoS_2_/Ge three-layer stacked structure (Fig. 11d)^30^. The indirect contact layer in MoS_2_/Ge forms a GeO_x_ barrier between the two materials, generating a dual-band tunable effect for visible and near-infrared detection. The BP/MoS_2_ layer on top provides mid-infrared response. By using both vertical and planar electrodes, the response magnitude of all three colors can be controlled (Fig. 11e). They also demonstrated the response behavior under three different working modes (Fig. 11f)^30^.

To develop more complex electrically tunable spectral shifting modes, nonlinear electrical modulation has been employed, enabling precise spectral discrimination^219^. 2D materials provide highly tunable functions beyond the constituent materials, paving the way for more complex spectral response variations^8^. These reconstruction-based computational spectroscopy techniques primarily rely on the encoding information obtained from the input light, which is then decoded and ultimately used to reconstruct the input spectral information^10^. Typically, this process includes three steps: learning, sampling, and reconstruction. Drawing inspiration from quantum dots (QDs)^228,229^, and CdSe_x_S_1-x_ nanowire spectrometers^230^, 2D materials have facilitated the development of a series of ultra-compact, novel computational spectrometers, covering wavelengths from visible to mid-infrared, such as BP^231^, ReS_2_/Au nanoparticles/WSe_2_^93^, MoS_2_/WSe_2_^232^, ReSe_2_/SnS_2_^233^, BP/MoS_2_^234^, and GeSe/InSe^235^. In pioneering works, the Stark effect observed in monolithic materials enabled electrically tunable nonlinear band splitting and displacement effects, which were considered prospective for spectral resolution applications. Yuan et al. used the Stark effect in BP to achieve gate voltage-controllable spectral response, leading to the first miniature mid-infrared (2-9 μm) spectrometer with a size of only 9 × 16 μm^2^ and a resolution of 40 nm^231^. Additionally, a homojunction PIN device based on single-component WSe_2_ was constructed, leveraging the nonlinear characteristics of a memristor to achieve high-performance spectroscopic resolution in the visible range, reaching up to 2 nm^236^. In addition to the aforementioned nonlinear effects in single-component materials that can generate additional equivalent spectral detectors, the band-to-band transition effect occurs at heterojunction interfaces. This intermaterial transition takes place between two coupled materials where their band structures hybridize, enabling partial charge carriers to transit from the valence band of one material to the conduction band of the other. Notably, this newly formed intermaterial bandgap is electrically tunable, thereby facilitating spectral resolution capabilities. In 2022, Deng et al. developed the first band-to-band transition near-infrared (1150-1470 nm) spectrometer based on van der Waals heterojunctions, enhanced by Au nanoparticles, achieving an optimized resolution of 20 nm^93^. The same year, Yoon et al. used the bipolar transfer characteristics of MoS_2_/WSe_2_ heterojunctions to achieve precise band bending and a spectral resolution of 3 nm in the visible range (405-845 nm)^232^. In 2024, they optimized the three-port spectrometer mentioned above, using the BTBT effect to construct a simplified dual-port BP/MoS_2_ heterojunction spectrometer^234^. Subsequently, Wu et al. utilized the interface-bound states of 2D ReSe_2_/SnS_2_ heterojunctions combined with band gate control to develop a spectral detector with non-volatile memory, covering 400–800 nm with a resolution of 5 nm^233^. To further enhance the expanded matrix dimensionality of miniaturized computational spectrometers, researchers have achieved this by incorporating additional information devoid of linear correlations. Studies have revealed that the floating MoS_2_ structures exhibit substantial electrostrictive effects. Through strain engineering, both photosensitivity and relaxation time can be simultaneously modulated, thereby extending additional matrix dimensions free from multicollinearity (Fig. 11g)^237^. By adding time-domain filtering and combining neural network algorithms, they achieved ultra-high precision ~1.2 nm wavelength resolution (Fig. 11h). This dual signal response characteristic induced by electrostriction provides a technical foundation for future realization of ultra-high resolution in ultra-compact devices.

However, current computational spectrometers, while theoretically utilizing coherent multi-band tunable spectral properties, typically have a limited spectral tuning range. This range often falls short of the required coverage, and even in the mid-infrared range, the resolution remains relatively low, leading to the potential loss of some spectral features.

## Mixed multi-dimensional detectors

After the detailed introduction of the six novel dimensional detectors, the key technical challenge lies in how to capture different dimensional information and systematically extract and classify these dimensional details within a unified framework. In general, whether through engineering, physics, or optical methods, it is essential to expand the response vector solution space to enable a more comprehensive and precise mapping of multidimensional information (including both linear and nonlinear techniques). Furthermore, nonlinear methods encompass certain information data sets that humanity cannot yet fully comprehend. These data sets often require advanced neural network algorithms to construct effective mappings, which offer substantial opportunities for reconstructing such information. By leveraging these algorithms, we can tap into the potential for greater understanding and efficient utilization of multidimensional information, facilitating the development of more sophisticated multidimensional fusion detectors.

### Multi-dimensional strategy and model

**Fig. 12:**
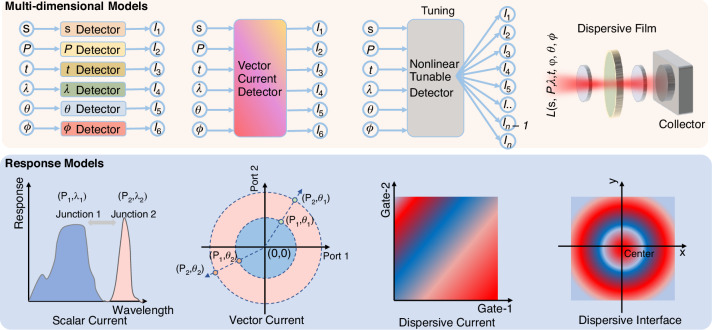
Multi-dimensional models

Based on current research, we have summarized the corresponding multidimensional design models and the representation of the final response data to better understand this cutting-edge technology (Fig. 12). There are currently four technical directions, some of which have been discussed in Fig. 4.

The linear responses of traditional single PN junctions can no longer support the expansion to more complex dimensions. While increasing sub-pixels or stacking multiple linear PN junctions can extend the capabilities of multidimensional detectors, these detectors typically face challenges with larger sizes and increased packaging complexity. On the other hand, vector photocurrent detectors can achieve a unified time-space response, outputting independent photocurrent responses at multiple ports, thus enabling the expansion of the solution space dimensions. The first two types of detectors are understandable in the context of current human knowledge, as they involve relatively straightforward physical processes.

With the introduction of nonlinear responses in 2D materials, it is possible to correlate physical quantum states with multidimensional light fields. By adjusting nonlinear quantum dots and utilizing gate control, one can achieve expansion of the solution space dimensions. Additionally, the dispersion behavior of materials can be mapped to multidimensional information, with the spatial distribution of light dispersion forming an analytical “code” for encoding information. These last two types of information require neural network algorithms for learning, allowing the reconstruction and restoration of the input multidimensional light field information.

### Existing mixed two-dimensional detectors

**Fig. 13:**
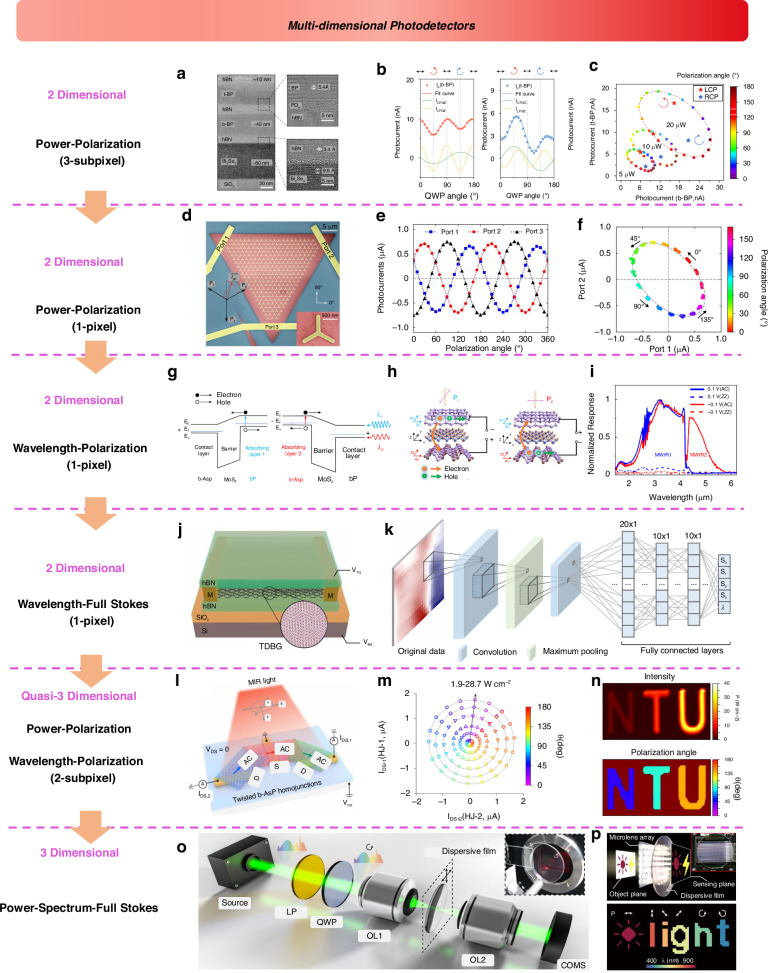
**Mixed Multi-dimensional detector**. Two-dimensional detectors; **a** Stacking BP/h-BN/BP/h-BN/Bi_2_Se_3_ framework^50^. **b** Linear polarization and circular polarization detection^50^. **c** Two ports output of the device^50^. Reproduced with permission^50^; Copyright 2022, American Association for the Advancement of Science. **d** Graphene/metasurface with three ports output^51^. **e** Time resolved photocurrents at three ports^51^. **f** The 2D plot with two ports as function of the polarization angle^51^. Reproduced with permission^51^; Copyright 2020, Nature Publishing Group. **g** Wavelength-dependent energy bands^238^. **h** Polarization related charge transfer conditions^238^. **i** The output polarization-spectrum at ±0.1 V^238^. Reproduced with permission^238^; Copyright 2024, Nature Publishing Group. **j** Twisted bilayer graphene with dual gate^37^. **k** Neural network algorithm learns and reconstructs polarization-wavelength information^37^. Reproduced with permission^37^; Copyright 2022, Nature Publishing Group. **Third-dimensional detectors**; **l** Double twisted Asp homojunction with three output ports^239^. **m** The two ports output various with different polarization angles^239^. **n** The multidimensional image demonstration^239^. Reproduced with permission^239^; Copyright 2024, Nature Publishing Group. **o** The optical dispersion assisted third-dimensional detectors based on lens-film-CMOS system^240^. **p** Demonstration of complex multi-dimensional imaging resolution^240^. Reproduced with permission^240^; Copyright 2024, Nature Publishing Group

To realize multidimensional fusion detectors, researchers have begun by integrating scalar power detection with other dimensions. Researchers have progressed from designing simple, constrained two-dimensional detectors to more advanced and mature two-dimensional detection systems, primarily focusing on polarization-power and polarization-single wavelength integrations. In the spatial division strategy, Xiong et al. achieved multidimensional detection within a single pixel (comprising three sub-pixels) using a stacked structure of two rotationally misaligned BP layers and an isotropic Bi_2_Se_3_ layer separated by h-BN (Fig. 13a)^50^. The isotropic Bi_2_Se_3_ served as a power calibration device, while the two BP layers with differing orientations allowed for the resolution of linear polarization angles. Furthermore, the device exhibited circular polarization discrimination due to symmetry breaking induced by Schottky barriers (Fig. 13b). Consequently, dual-terminal optical responses enabled the distinction of power, linear polarization, and circular polarization (Fig. 13c), though the device could not fully resolve elliptical polarization, potentially leading to ambiguity in solutions. Wei et al. optimized the device structure by integrating metasurfaces with graphene (Fig. 13d), achieving a unified time-space detection device^51^. The artificial photovoltaic effect in the constructed system enabled the independent output of two degrees of freedom through vector photocurrent at three ports, adhering to Kirchhoff’s circuit laws (Fig. 13e). Using a concentric circular design strategy with the PR of -1, the device could effectively distinguish power and linear polarization (Fig. 13f)^51^. Beyond power-polarization fusion, researchers have explored integrating polarization with spectral dimensions for more complex device designs. Zhang et al. utilized an asymmetric single-barrier structure (Asp/MoS_2_/BP, Fig. 13g, h) to construct back-to-back heterojunctions with distinct response wavelengths and initial phase angles^238^. This configuration achieved wavelength and polarization state differentiation, with a division point at a mid-infrared wavelength of 4.2 μm under ±0.4 V bias (Fig. 13i)^238^. To advance beyond linear polarization detection and achieve full-Stokes polarization detection, further optimization of device structures is necessary. Ma et al. used 1.2° twisted bilayer graphene to map topological quantum states to input wavelengths and full-Stokes polarization states (Fig. 13j)^37^. By modulating the top and bottom gate voltages, they obtained a bipolar response map associated with optical responses (Fig. 13k). Through neural network learning of this bipolar photocurrent feature, the device could reconstruct unknown full-Stokes polarization states and wavelengths (Fig. 13k), marking a significant advancement in multidimensional polarization-spectral fusion detection^37^.

### Existing mixed third-dimensional detectors

The aforementioned 2D detectors primarily rely on single wavelength or power as the foundational constraint dimensions, which are treated as simple scalars. This limitation naturally leads to two-dimensional detection systems. However, advancing toward fully integrated three-dimensional detection poses significant challenges. The structural design and response mechanisms result in overlapping data from different dimensions, making it difficult to disentangle and recombine this information effectively within the detector itself. This issue is especially pronounced in low-pixel or single-pixel devices, where achieving the functionality of multi-pixel systems requires electrical modulation, often leading to crosstalk and reducing reconstruction accuracy. To overcome these limitations and move toward quasi-three-dimensional or fully three-dimensional detectors, more complex optical and physical response models that generate nonlinear solution spaces are required. Researchers have made strides in this direction. In early 2024, Wang et al. developed a double twist BP device^239^. This device features two homojunction BP segments with 135° rotation, leveraging polarization-induced PTE distribution and bipolar characteristics (Fig. 13l)^239^. The device functions as a dual-output bipolar linear polarization detector with distinct initial phase angles and a polarization ratio (PR) of -1. Previous dual-terminal devices struggled to provide unique solutions due to overlapping focal points caused by different power levels and polarization angles. In Wang’s device, however, the output current characteristics form curves whose centers remain fixed at the origin under specific power conditions (Fig. 13m), ensuring unique solutions for different power levels and polarization states. Furthermore, the device achieves unique solutions for monotonic wavelength-polarization state detection. It also demonstrated effective reconstruction of multidimensional input data, such as alphabet-like patterns, achieving a quasi-three-dimensional photodetector (Fig. 13n). To further mature multidimensional detectors, Fan et al. developed a simple imaging optical system comprising a prism, thin film, and CMOS sensor (Fig. 13o)^240^. This system encodes multidimensional optical information into a single image, capturing specific polarization and wavelength dispersion details. Using a deep residual network, the encoded data represented as azimuthal and incident angles in the image was reconstructed to yield full-Stokes and spectral information (Fig. 13p)^240^. The relative power in the spectral resolution part may require additional calibration to achieve complete three-dimensional information resolution.

This approach demonstrates the capability to handle complex multidimensional input while offering a compact design compatible with industrial camera sensor manufacturing. These advancements provide valuable insights for developing the next generation of multidimensional devices, combining compactness, efficiency, and compatibility with existing industrial standards (Tables 1–6).

**Table 1:** The performance of intensity detectors

Framework	Mechanism	DR Condition	Wavelength	Responsivity	Speed	Ref.
MoS_2_/Gra/WSe_2_	P-G-N sandwich	Purely linear	2400 nm	4250 A/W	53.6 μs	^90^
MoS_2_/MoTe_2_	Interlayer transitions	Purely linear	1550 nm	~1 nA/W	~50 ms	^92^
BP/Bi_2_O_2_Se	Momentum-matched interband transitions	Purely linear	3600 nm	1.22 A/W	118 μs	^94^
Gra/PbS QDS	Photogating effect	Sublinear	1450 nm	10^7 ^A/W	10 ms	^98^
MoS_2_/WSe_2_	Photogating effect	Sublinear	800 nm	2700 A/W	17 ms	^87^
WSe_2_/Ta_2_NiSe_5_	Photogating-assisted tunneling effect	Sublinear	1550 nm	7300 A/W	1 μs	^99^
Ta_2_NiSe_5_	Distinctive recombination centers	Superlinear	808 nm	17.21 A/W	3 s	^104^
Ta_2_NiS_5_	Distinctive recombination centers	Superlinear	632 nm	2.5 mA/W	31 μs	^105^
Gra/SiO_2_/Si/Gra	Hot carriers photothermionic effect	Superlinear	3800 nm	0.1 A/W	<1 μs	^102^
3R bilayer MoS_2_	BPVE	Linear to square root	532 nm	1.5 mA/W	NA	^113^
ReS_2_/ReS_2_	BPVE	Linear to square root	VIS	~1.5 μA/W	NA	^110^
MoS_2_/BP	Dual polarization BPVE	Linear to square root	633 nm	~2 mA/W	2.2 ns	^116^

**Table 2:** The performance of the spatial detector

Framework	Mechanism	Function	Wavelength	Sensitivity	Speed	Ref.
Gra/Si	Schottky response	x-y detection	1550 nm	43 mV/mm	0.44 µs	^127^
Gra/Ge	Schottky response	x-y detection	1600 nm	47 mV/mm	10 µs	^128^
Gra/p-Si/n-Si	Schottky-PN junction	x-y detection	1100 nm	~40 mV/mm	16 µs	^129^
WSe_2_	Bipoar PC	On-chip perception	522 nm	60 mA/W	50 ns	^132^
WSe_2_/hBN/Al_2_O_3_	Interface floating gate	Spatial operation (Laplacian operator)	650 nm	>100 A/W	8 ms	^136^
WSe_2_/h-BN/Al_2_O_3_ with Pt/Ta/HfO_2_/Ta	Interface floating gate	Spatial operation with a memristor	650 nm	>100 A/W	<10 ms	^137^
PdSe_2_/MoTe_2_	Bipoar PV	Spatial operation (Sober operator)	980 nm	320 A/W	400 ns	^138^
Gra/Ge	PV effect	Spatial operation (dynamic convolution)	1550 nm	~0.1 A/W	25 µs	^139^

**Table 3:** The performance of temporal detector

Framework	Mechanism	Function	Wavelength	Responsivity	Speed	Ref.
hBN/WSe_2_/Al_2_O_3_/BP	Bipolar memory	Motion detection	700 nm	146 A/W	100 µs	^141^
Rippled-assisted MoS_2_	Bipolar memory	Motion detection (all day condition)	940 nm	181 A/W	1 ms	^142^
MoS_2_ with trap	Graded memory	Motion detection (moving state)	660 nm	~100 mA/W	10–10^6 ^ms	^143^
Gra/SiO_2_/i-Si	Bipolar Spike	Event-driven motion detection	685 nm	~1 mA/W	10 ms	^145^
Homojunction WSe_2_	Bipolar Spike	Event-driven motion detection with synapse	660 nm	75 mA/W	5 µs	^144^
GeSe/WSe_2_	Bipolar Response	Motion detection	532 nm	~10 μA/W	NA	^250^

**Table 4:** The performance of polarization detector

Framework	Mechanism	Function	PR or g factor	Wavelength	Responsivity	Speed	Ref.
Bp	PC, Anisotropy	LP detection	3	3.7 µm	0.35 mA/W	40 μs	^159^
WSe_2_/ReSe_2_	PV, Anisotropy	LP detection	2	980 nm	0.57 A/W	2 μs	^168^
ReS_2_/In_2_Se_3_	PV, Ferroelectric anisotropy	LP detection	2.5	1064 nm	2.21 A/W	120 μs	^169^
Bp/InSe	PV, anisotropy	LP detection	10.76	633 nm	10^5 ^A/W	24 ms	^176^
PdSe_2_/Graphene	PTE, Anisotropy	LP detection	1.21	10.5 µm	13 V/W	253 μs	^69^
BP/MoS_2_/Graphene	Unipolar barrier, Anisotropy	LP detection	4.9	3.7 µm	1.8 A/W	23 μs	^27^
WSe_2_/Ta_2_NiSe_5_/WSe_2_	Fully Depleted, Anisotropy	LP detection	14.8	1550 nm	0.436 A/W	420 μs	^183^
MoSe_2_/GeSe/MoSe_2_	Fully Depleted, Anisotropy	LP detection	12.5	635 nm	0.206 A/W	5.19 ms	^185^
hBN/Asp/hBN	PC, Anisotropy	LP detection	14	7.7 µm	1.2 mA/W	500 μs	^188^
Graphene/PdSe_2_/Ge	PV, Anisotropy	LP detection	112	650 nm	0.3 A/W	92.5 μs	^187^
Bp/MoS_2_/Bp	Back-to-back junctions	LP detection	100	3.7 µm	0.9 A/W	3.7 μs	^186^
Te	Anisotropy	LP detection	9	3 µm	1.36 × 10^3 ^A/W	62.7 μs	^67^
Nb_2_GeTe_4_/MoS_2_	Anisotropy	LP detection	-3.38 and 48	11 µm	1.59 mA/W	253 μs	^73^
1T’-MoTe_2_/WSe_2_	Anisotropy, Schottky	LP detection	1 → + ∞ / − ∞ → − 1 (LP)	635 nm	0.212 A/W	117 μs	^191^
CdSb_2_Se_3_Br_2_/WSe_2_	Bipolar bulit-in field	LP detection	1 → + ∞ / − ∞ → − 1 (LP)	532 nm	NA	NA	^251^
2H-MoTe_2_/PVDF	Ferroelectric BPVE	LP detection	1 → + ∞ / − ∞ → − 1 (LP)	1550 nm	20 mA/W	NA	^252^
Graphene/Heterogeneous metasurface	Vector Current	LP detection	1 → + ∞ / − ∞ → − 1 (LP)	4 µm	15.6 V/W	667 ns	^58^
2D COFs	Chiral materials	CP detection	0.5	405 nm	1.05 A/W	26.4 ms	^192^
MoS_2_	Valley-associated inequivalent excitations	LP, CP detection	1.2 (LP), 0.02 (CP)	670 nm	0.28 A/W	33 ms	^194^
Te	CPGE	CP detection	0.3	10.6 µm	150 μA/W	NA	^79^
PdSe_2_/2H-MoTe_2_	Spin Hall effect	LP, CP detection	2 (LP), 0.01 (CP)	2200 nm	7.3 × 10^3 ^A/W	45 μs	^80^
Graphene/centrosymmetric metasurface	Vector Current	Pure CP detection	84	4 µm	98 V/W	886 ns	^34^
Asp/WSe_2_/Asp	Back-to-back junctions	LP state	55	4.6 µm	6.74 μA/W	200 μs	^49^
PdSe_2_/3 pixel Chiral metasurface	PTE	Full Stokes	1→+∞/−∞→−1 (both CP and LP)	7 μm	3.6 V/W	76 μs	^196^
MoS_2_/4 pixel metasurface	4 Scalar plasmon	Full Stokes	100 (Extinction ratio)	1.6 μm	~100 μA/W	19.5 μs	^197^

**Table 5:** The performance of phase detector

Framework	Mechanism	Function	Wavelength	Sensitivity	Speed	Ref.
WTe_2_/“U” shaped electrode	OPGE	Topological charge of OAM	1000 nm	NA	Scan CPL (>1 s)	^52^
TaIrTe_4_/“U” shaped electrode	OPGE	Topological charge of OAM	4000 nm	j × 14.4 nA/W	Scan CPL (>1 s)	^53^
Multiplexing nanoring slits	SPP	Topological charge of OAM	700 nm	selectivity ≥ 0.1	NA	^206^
PdSe_2_/Spin Hall Coupler	PTE and SPP	Topological charge and Polarization states	8000 nm	j × 0.2 nA/W	69 μs	^207^
Graphene/“U” shaped electrode	OPGE	Topological charge of OAM	4000 nm	24.7 μA/W	Scan CPL (>1 s)	^208^

**Table 6:** The performance of spectral detector

Framework	Mechanism	Port	Footprint	Wavelength	Resolution	Ref.
BP/MoS_2_/p-Si	Back-to-back junctions	Two	20 × 20 μm^2^	NIR-MIR	Dual-band	^31^
BP/MoS_2_/MoTe_2_	Back-to-back junctions	Two	~50 × 80 μm^2^	NIR-MIR	Dual-band	^224^
BP/MoS_2_/PbS QDs	PC/PV switching	Two	~15 × 30 μm^2^	NIR-MIR	Dual-band	^225^
MoS_2_/Ge	Band-to-band tunneling	Two	10 × 10 ×${\rm{\pi }}$ μm^2^	VIS-NIR	Dual-band	^226^
BP/MoTe_2_	Energy band modulation	Two	~12 × 12 μm^2^	VIS-NIR-SWIR-MWIR	Mixed-band	^227^
BP/MoS_2_/Ge	Junctions control	Three	20 × 20 μm^2^	VIS-SWIR-MWIR	Three-band	^30^
BP	Stark effect	Four	9 × 16 μm^2^	2000–9000 nm	40 nm	^231^
ReS_2_/Au NPs/WSe_2_	Interlayer transition	Three	20 × 20 μm^2^	1150–1470 nm	20 nm	^93^
MoS_2_/WSe_2_	Energy band bending	Three	22 × 8 μm^2^	405–845 nm	3 nm	^232^
ReSe_2_/SnS_2_	Trap state control	Three	19 × 19 μm^2^	400–800 nm	5 nm	^233^
BP/MoS_2_	Band-to-band tunneling	Two	30 × 20 μm^2^	500–1600 nm	2 nm	^234^
GeSe/InSe	Voltage-tunable band alignment	Two	25 × 25 μm^2^	400–1100 nm	0.35 nm	^235^
MoS_2_	Electrostrictive effect	Three	20 × 25 μm^2^	400–860 nm	1.2 nm	^237^
PIN-WSe_2_	Nonlinear memristor	Two	4 × 9 μm^2^	400–800 nm	2 nm	^236^

### Performance metrics and summary of multi-dimensional detectors

**Fig. 14:**
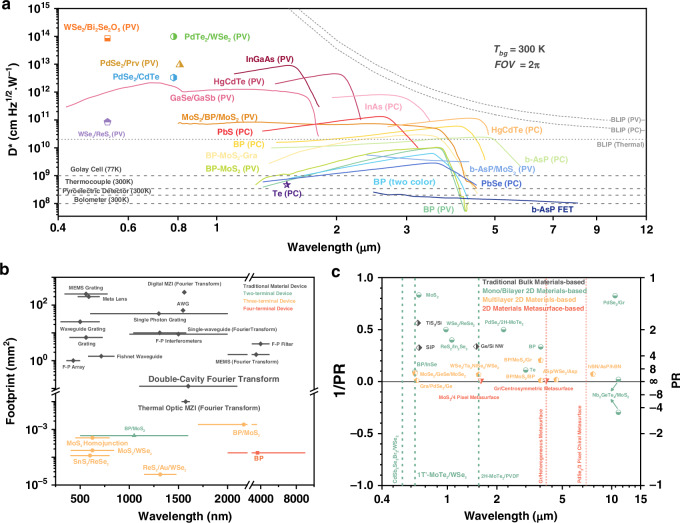
**Performance comparison with traditional materials detection devices**. **a** The sensitivity of 2D based multi-dimensional detectors^27,31,70,159,160,174,186,254–261^. **b** The spectral detector^33,93,231–234,237,262–273^. **c** The polarization detectors^27,49,58,67,69,73,80,159,168,176,183,185–188,191,194,196,197,251,252,274–276^

The development of multidimensional detectors has progressed from initial two-dimensional exploration to the emergence of relatively mature three-dimensional devices, revealing technical challenges associated with multidimensional information integration. We summarize the performance and technical metrics of multidimensional detectors in Table 7 and performance comparison with traditional material detection devices (Fig. 14). The sensitivity of some multidimensional detectors based on two-dimensional materials has already surpassed that of traditional bulk materials. However, challenges related to fabrication stability and large-area scalability remain to be addressed. Moreover, a unified evaluation framework or quality factor for comparing relevant parameters is still lacking. Through comparison, we observe that most existing multidimensional technologies utilize effects such as PTE effect, BPVE, and PV mechanisms. These approaches result in minimal energy consumption and high response speeds, often meeting MHz bandwidth requirements, paving the way for practical applications. Additionally, novel evaluation metrics, such as crosstalk and reconstruction accuracy, are not universally applicable, with only a few studies mentioned them. 2D material-based tilt structures and channel advantages, often combined with metasurface designs, have been widely used in multidimensional detection. However, these approaches are largely confined to the polarization-spectral domain due to their dependence on quantum states and vibrational modes. Among the six possible dimensions, significant potential remains for integrating additional resolution dimensions. Currently, most technologies are limited to power, spectrum, and polarization dimensions. Integration of other dimensions has yet to achieve a breakthrough. By leveraging narrow-bandgap materials like BP and graphene, along with mechanisms such as PTE and polaritons, the spectral range has been extended into the MIR region. Existing studies can now cover the entire 3-8 µm range, highlighting the promise and challenges of multidimensional detection technologies.

**Table 7:** The performance of mixed multi-dimensional detectors

Framework	Mechanism	Strategy	Power	Intensity	Spectral	Polarization	Wavelength	Speed	Ref.
Twisted Bp-Bi_2_Se_3_	Schottky symmetry breaking	Divided Space (3 pixels stack)	0-0.01 V	✓	⍰	✓(LP and CP)	1.55 µm	800 ns	^50^
Graphene\“T” shaped metasurface	Artificial BPVE	Time-Space Unified	0 V	✓	⍰	✓(LP)	4 µm	100 µs	^51^
Bp\MoS_2_\Asp	Back to back junctions	Divided Time (2 pixels stack)	$\pm$0.1 V	⍰	✓	✓(LP)	2–5.5 µm	500 ns	^238^
Graphene/Dual-arm Metasurface	Artificial BPVE	Time-Space Unified	0 V	⍰	✓	✓(LP and CP)	1.5–8 µm	2.8 µs	^253^
Twisted Bilayer Graphene	BPVE, Topological quantum states	Divided Time	0 V	⍰	✓(Wavemeter)	✓(Full Stokes)	5.0–7.7 µm	<50 µs	^37^
Double Twisted Asp	PTE	Divided Space (2 pixels)	0 V	✓	✓(Wavemeter)	✓(LP)	3.7–5.7 µm	21 µs	^239^
Lens-Film-CMOS	Dispersion distribution	Optical System	Others	✓	✓	✓(Full Stokes)	0.4–0.9 µm	NA	^240^

## Future development and way to on-chip integration

Due to current technological constraints, multidimensional composite detectors are limited to resolving three dimensions. To extend detection capabilities to additional dimensions, it is necessary to exploit nonlinear optical properties of two-dimensional quantum materials, combined with sub-pixel stacking engineering. These approaches can generate more complex nonlinear vector equations for dimensional resolution. However, whether achieved through optical or physical mechanisms, some composite outputs remain challenging for humans to interpret. Advanced neural networks will be indispensable for establishing unknown one-to-one mappings, learning, and ultimately reconstructing the multi-dimensional information encoded in these outputs. Future research should also focus on defining the upper and lower limits of parameters required for multidimensional detectors. This will enhance our understanding of the relationships and interactions among multi-dimensional information channels. The inherent compactness of 2D materials provides a significant advantage for the on-chip integration of multi-dimensional detectors. Such integration is crucial for developing next-generation devices capable of simultaneous multidimensional detection and processing within a unified platform. This progress will pave the way for practical, high-performance multi-dimensional photodetectors tailored for complex real-world applications.

### Multi-parameter detectors and multi-dimensional detectors

**Fig. 15:**
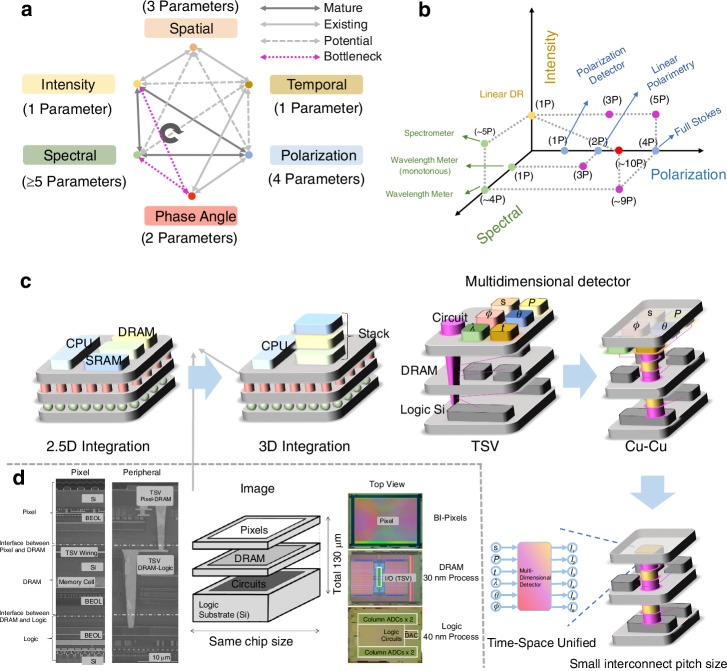
**Multi-parameter characterization for plenoptic-field detectors and advanced integration technologies of multi-dimensional detector**. **a** The relationship between multiple dimensions. **b** The specific development of the power-polarization-spectrum part. **c** The optimization of interconnect pitch size in multi-dimensional detectors. **d** Stacked backside illuminated-CMOS image sensor by Sony 3D integration technology^248^. Reproduced with permission^248^; Copyright 2009, IEEE

From the perspectives of engineering feasibility and industrial maturity, single-dimensional multivariate detectors are more practically applicable. This is because they can be adapted using existing detectors and optical systems, and the supporting technologies are relatively mature. Moreover, by focusing on a single dimension, the system design can be more easily optimized. In contrast, multidimensional fusion detectors represent a more cutting-edge field. Multimodal recognition requires higher precision and better robustness to capture complex, multidimensional optical features. This often involves multimodal coupling devices, complex synchronization mechanisms, and extensive data processing. Figure 15a, b illustrates the relationship between single-dimensional multivariate detectors and multidimensional multivariate detectors. In Section 4, we provide a detailed introduction to six types of detectors. Among them, relatively mature single-dimensional multivariate detectors include full-Stokes polarization detectors, spectrally resolved detectors, and motion detectors. Due to current technological limitations, multidimensional multivariate detectors often require certain constraints on multidimensional information resolution (e.g., power-spectrum-polarization). In the future, users can choose between single-dimensional and multidimensional multivariate detectors based on application scenarios, system complexity, and resource constraints.

Current multidimensional photodetectors face trade-offs between temporal and spatial dimensions, just enabling single-pixel three-dimensional detection (Fig. 15a). In contrast, vector photocurrent detectors, which unify temporal and spatial domains, are presently limited to 2D detection under constrained conditions. Among these, intensity-spectrum-polarization detectors have seen significant advancements. Utilizing well-developed materials such as zero-bandgap graphene, black phosphorus, and topological quantum materials offers promising potential for fully integrated three-dimensional power-spectrum-Stokes parameter detectors in the mid-infrared region. Other dimensions, including polarization-phase angle and temporal-spatial fusion detectors, have shown preliminary promise but have not yet achieved full three-dimensional detection capabilities. Integration of phase angle with power and spectrum dimensions remains particularly challenging due to the reliance on amplitude measurements in phase angle detection. Phase angle detectors, particularly those designed for vortex light, inherently link temporal and polarization dimensions. They hold potential for multidimensional detection with the integration of advanced nanoantenna designs and novel 2D topological materials. Temporal-spatial dimension detectors employing 2D materials are currently confined to the visible and near-infrared wavelength ranges. This limitation arises because the predominant photoactive materials capable of wafer-scale epitaxial growth are restricted to transition-metal dichalcogenides (TMDs). Progress in this area requires the development of mid-infrared topological and thermoelectric materials to extend the wavelength range and enable sophisticated infrared motion tracking and spatial computation.

Focusing on the most mature dimensions (intensity, spectrum, and polarization) (Fig. 15b) highlights critical total parameter requirements. These fundamental investigations into the relevant parameters have also revealed the complexities involved in detecting multidimensional information of physical optical fields. We have annotated simplified dimensional detectors operating under three primary dimensions, along with the required independent input parameters for each corresponding dimension. Consequently, higher-dimensional detectors with elevated coordinate positions would demand additional parametric inputs. While intensity detection requires a single parameter, full-Stokes polarization detection demands four parameters, and spectral detection is even more complex, necessitating approximately five parameters for a complete spectral detector^241^. Consequently, a comprehensive three-dimensional detector would require ten input parameters, posing significant challenges for single-pixel devices and often resulting in temporal and spatial compromises. To achieve practical devices that unify temporal and spatial dimensions, future research should emphasize fast electrical modulation, structural optimization, exploration of novel physical mechanisms, and advanced neural network algorithms. These approaches could lead to multidimensional detectors with enhanced overall performance. Although detectors capable of unifying geometric and physical optical field domains for full optical-field information dimensionality are theoretically feasible, they would likely involve significant trade-offs in terms of accuracy, bandwidth, and other performance metrics. This is primarily because physical optical field dimensions represent oblique mappings of intensity, where substantial dimensional information exhibits considerable overlap, making crosstalk-free data separation challenging to achieve. As such, three-dimensional detectors represent a balanced solution for multidimensional detection systems, offering an optimal compromise between complexity and performance.

### New response mechanism

Currently, among the methods we have summarized, multidimensional detectors are primarily realized through four mechanisms: optical scattering, vector photoelectric current metasurfaces, back-to-back heterojunctions, and twisted-angle 2D heterostructures. To enable more complex and ultra-high-parameter multidimensional detection, more sophisticated physical and material mechanisms need to be proposed to keep pace with the current explosive development trends. For example, the recently reported unique Moiré scattering effects in Moiré photonic crystals have attracted widespread attention. In addition, microscopic modulation approaches in two-dimensional materials, such as strain and interlayer sliding, offering new opportunities for the expansion of multidimensional detection and hold great potential for translating fascinating optical physics into practical applications. Phenomena such as the valleytronic Hall effect and asymmetric band absorption provide novel strategies for the design of multidimensional detectors, opening new pathways for the development of higher-dimensional detection systems. However, it is equally important to address how these remarkable physical mechanisms can be harnessed to reconstruct multidimensional information with higher precision and better robustness.

### Environmental stability

Graphene, TMDs, and BP exhibit distinct humidity and oxygen sensitivities, rendering their optoelectronic properties incapable of maintaining high performance during prolonged operation in ambient atmospheric conditions. For certain 2D materials, moisture absorption leads to increased scattering, crystal structure degradation, unwanted doping, or phase transitions, which severely compromise the multi-dimensional parameter resolution capability of detectors. Consequently, nitrogen-environment encapsulation processes become essential to preserve their high-resolution performance in multi-dimensional detectors, including advanced techniques such as ultrafast laser sulfidation, molecular self-assembled monolayers, and hBN encapsulation. Through material modification, advanced packaging, and process optimization, the encapsulation standards for some materials have approached industrial requirements. However, sensitive materials like BP still require significant breakthroughs. Future developments must incorporate cross-scale design strategies (ranging from atomic-level defect control to macroscopic packaging) to achieve comprehensive stability enhancement.

### Large-area compatibility with standard CMOS processes

According to the aforementioned multi-dimensional technologies, there is still a long way to go before large-area fabrication can be fully realized. Many metasurfaces integrated with 2D materials have achieved multi-dimensional detection under vector photocurrent, choosing materials like graphene and MoS_2_ as the field collection layer, which are compatible with large-area fabrication. Currently, large-area wafer-scale fabrication of graphene and MoS_2_ has been demonstrated in electronic transistors via CVD and MOCVD techniques. However, metasurfaces still face significant challenges in large-area fabrication due to their complex processing and high costs, particularly in terms of fabrication precision. Moreover, the growth of twisted 2D materials is even more challenging, as it requires precise control over nucleation sites and twist angles. Achieving large-area, array-based multi-dimensional detectors still demands the implementation of co-design strategies between device systems and coordinated optimization of technological systems.

Even if the large-area fabrication challenges for 2D materials in multidimensional applications are resolved, their integration with standard CMOS processes still presents both challenges and opportunities. For instance, most two-dimensional materials require high-temperature CVD growth (~800–1000 °C), while the back-end-of-line (BEOL) CMOS processes impose a strict thermal budget of <450 °C. This necessitates the development of low-temperature CVD/PECVD techniques as potential solutions, although wet/dry transfer methods may introduce undesirable defects. Another significant technical hurdle involves the Fermi-level pinning effect at metal-2D material interfaces, which leads to high contact resistance. This issue may be addressed through in-situ growth phase engineering for contact optimization.

### On-chip and integration

To meet the demands of on-chip multi-dimensional detection with 2D materials, innovative integration strategies are essential to overcome the space limitations of traditional multi-dimensional detectors. Conventional spatial division approaches require substantial space, but the compact nature of 2D materials provides an opportunity to rethink integration techniques. With the transition from FinFET to GAAFET and MBCFET architectures^242,243^, individual transistors are constrained by Moore’s Law, locked at a 2 nm node, making further chip size reductions challenging^244^. The integration methods fundamentally influence chip energy efficiency and integration density. Early 2.5D planar interconnection methods evolved into tightly bonded strategies with interposers called “chip on wafer on substrate” (Fig. 15c), optimizing the spatial arrangement of different functional chips^245^. Wafer-to-wafer bonding technology facilitates 3D stacking integration, significantly increasing interconnection density and reducing interconnection lengths^246^. While CPUs still face challenges in vertical stacking, image processors have benefited through-silicon via (TSV) technology. TSV allows for stacking detectors, DRAM, and logic components in a 3D architecture (Fig. 15d)^247,248^.

Advancements such as wafer-to-wafer Cu-Cu bonding further compress the interconnection size to as small as 100 nm, providing an advantage for compact multi-dimensional detectors^249^. Multi-dimensional fusion detectors based on 2D materials exhibit compactness, precision, and high integration potential^130^. Ultrathin 2D materials enable tighter 3D integration stacks while addressing the heat dissipation challenges inherent in such architectures. The superior thermal conductivity of 2D materials offers a promising solution to thermal management in densely stacked 3D designs. The compatibility of 2D material processes with existing Si-based fabrication techniques requires further exploration. A compact, multi-dimensional detector eliminates the need for focal plane segmentation. Single-unit devices unifying spatial and temporal dimensions have the potential to enable more compact, high-resolution image sensors for practical applications.

### Future applications

**Fig. 16:**
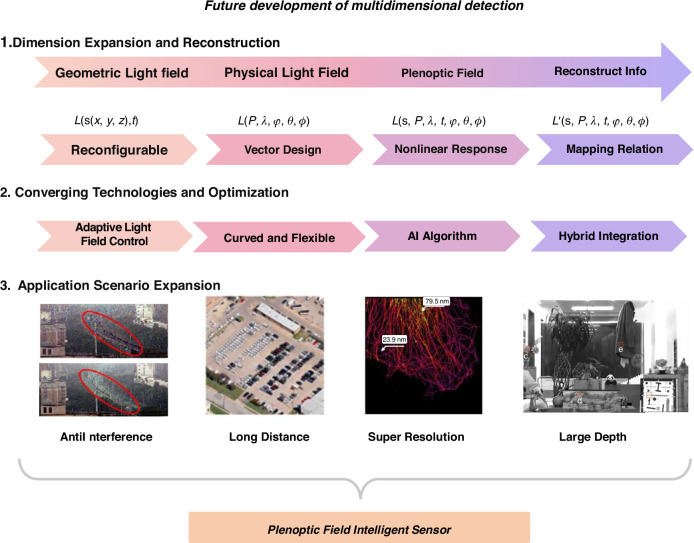
**Future development of multi-dimensional detectors, anti interference**^277^, **long distance**^278^, **super resolution**^279^, **large depth**^280^. Reproduced with permission^277^; Copyright 2024, Optica Publishing Group. Reproduced with permission^278^; Copyright 2022, Wiley-VCH. Reproduced with permission^279^; Copyright 2023, Nature Publishing Group. Reproduced with permission^280^; Copyright 2022, Nature Publishing Group

Multidimensional detectors capable of simultaneously resolving intensity, polarization, spectral, and phase information are expected to play a transformative role across a wide range of emerging technologies. As detection evolves from geometric light field resolution to physical light field resolution and ultimately toward full light field information capture (enabled by the adaptive light field control, AI-driven algorithms, curved device configuration, and heterogeneous integration), the potential for higher imaging resolution, smaller system form factors, and longer detection ranges is significantly expanded (Fig. 16). This progression is poised to trigger a new wave of applications in optical imaging^130^.

Future implementations may include adaptive and interference-resistant imaging (e.g., defogging and dehazing), ultra-long-range imaging enhanced by polarization-based contrast mechanisms, wide-area super-resolution imaging for biomedical applications, and extended depth-of-field imaging for enhanced depth perception (Fig. 16). In addition, quantum information processing and optical communication systems are likely to benefit from multidimensional encoding and detection, which exploit additional degrees of freedom in the light field to boost both information capacity and system security.

With the continued advancement of on-chip photonic integration, compact and tunable multidimensional detectors are expected to become key components of next-generation platforms in computational optics, machine vision, and AI-assisted imaging^10^. The core value of their future applications lies in enabling higher-dimensional, more precise, and real-time data fusion, thereby facilitating deeper and more comprehensive understanding of complex systems, ranging from microscopic particles and the human body to machines, environments, and even astronomical phenomena.

## Conclusion

The rise of multidimensional detectors leveraging 2D materials heralds a new era of low-power, low-circuit-complexity, and functionally enriched vision sensors. This article provides an overview of six novel types of dimensional detectors and the current state of multidimensional fusion detectors. Ongoing advancements in material engineering, integration technologies, and computational methods promise to overcome current limitations, paving the way for a new paradigm in low-power, multifunctional vision sensing. With continuous advancements in 3D integration and 2D material engineering, the realization of highly integrated, efficient, and versatile multi-dimensional detection systems is on the horizon. These technologies will redefine the landscape of high-performance image sensors, offering unprecedented precision and compactness for next-generation applications.
